# The impact of gait pattern personalization on the perception of rigid robotic guidance: a pilot user experience evaluation

**DOI:** 10.3389/frobt.2026.1937649

**Published:** 2026-10-30

**Authors:** Beatrice Luciani, Katherine Lin Poggensee, Heike Vallery, Alex van den Berg, Severin David Woernle, Mostafa Mogharabi, Stefano Dalla Gasperina, Laura Marchal-Crespo

**Affiliations:** 1 Cognitive Robotics Department, Delft University of Technology, Delft, Netherlands; 2 Department of Rehabilitation Medicine, Erasmus Medical Centre, Rotterdam, Netherlands; 3 Department of Biomechanical Engineering, Delft University of Technology, Delft, Netherlands; 4 Institute of Automatic Control, RWTH Aachen University, Aachen, Germany; 5 Department of Mechanical Engineering, FUM Center of Advanced Rehabilitation and Robotics Research (FUM CARE), Ferdowsi University of Mashhad, Mashhad, Iran; 6 Walker Department of Mechanical Engineering, University of Texas at Austin, Austin, TX, United States

**Keywords:** exoskeleton, gait pattern personalization, gait rehabilitation, human-centered robotics, kinematics

## Abstract

**Introduction:**

Robot-aided gait rehabilitation facilitates high-intensity and repeatable therapy. However, most exoskeletons rely on pre-recorded, non-personalized gait trajectories constrained to the sagittal plane, potentially limiting movement naturalness and user comfort.

**Methods:**

We present a data-driven gait personalization framework for an exoskeleton that supports multi-planar motion, including hip abduction/adduction and pelvic translation and rotation. Personalized trajectories to individual participants were generated using regression models trained on anthropometric, demographic, and walking speed data from a normative database. In a within-subject experiment involving ten young unimpaired participants, these personalized trajectories were evaluated in regard to comfort, naturalness, and overall experience and compared against two standard patterns from the same database: one averaging all the trajectories, and one randomly selected.

**Results:**

We did not find significant differences across pattern conditions, despite all trajectories being executed with high accuracy thanks to a stiff position-derivative controller. We found, however, that pattern conditions in later trials were rated as significantly more comfortable and natural than those in the first trial.

**Discussion:**

Our results suggest that participants might have adapted to walking within the exoskeleton, regardless of the enforced gait pattern, and highlight the importance of integrating subjective feedback when designing personalized gait controllers and accounting for user adaptation during experimentation.

## 1 Introduction

Lower-limb exoskeletons are widely used in gait rehabilitation to deliver high-intensity, task-specific training while reducing therapists’ physical burden Moucheboeuf et al. (2020); Chen et al. (2016). A variety of robotic devices have been developed for gait training, such as the Lokomat Marchal-Crespo and Riener (2022), LOPES Meuleman et al. (2016), ABLE ?, Hayes et al. (2022), AIDER Zou et al. (2021), and TWIN exoskeletons Vassallo et al. (2020). These exoskeletons can implement a variety of control modalities to either enhance the device’s responsiveness and adaptation to human movement or more rigidly guide patients’ leg segments along predefined gait trajectories, exploiting trajectory-tracking control strategies de Miguel-Fernández et al. (2023); Marchal-Crespo and Reinkensmeyer (2009). Trajectory-based guidance is especially exploited to physically support more severely impaired patients who have minimal motor control.

A common assumption in rehabilitation is that training that feels natural and lifelike Cucinella et al. (2025) promotes the transfer of the acquired skill to the real world, in line with the specificity hypothesis Schmidt and Wrisberg (2008) Therefore, recent attempts have been made to realize robots that allow for more naturalistic walking, beyond supporting walking in the sagittal plane. For example, to reproduce the natural side-to-side shifts and pelvic rotations during gait, the most recent commercial versions of the Lokomat (from 2014 on) can incorporate lateral translation and transverse rotation of the pelvis through linear actuation on a new pelvis module, namely, the FreeD Module (Hocoma, Switzerland) Aurich-Schuler et al. (2019). Other solutions include actuated hip ab-/adduction, such as the LOPES II exoskeleton Meuleman et al. (2016), whose end-effector structure approach with parallel actuation further facilitates the alignment of human-robot joints. In general, the addition of passive/actuated degrees of freedom allows a more natural gait pattern, thereby exciting more physiologically-based sensory information from cutaneous, muscular, and joint mechanoreceptors and facilitating balance training.

Despite hardware advances, many current exoskeletons still enforce predefined gait trajectories based on pre-recorded data from unimpaired individuals (e.g., Yeung et al. (2017) or from therapist-guided training (e.g., Emken et al. (2008). Yet, every person’s gait pattern is unique Kale et al. (2004). Some of this inter-subject variability can be explained by individual-specific factors such as age, gender, and body measurements Ren et al. (2023), and gait speed Hanlon and Anderson (2006). Employing standard gait patterns can increase the usability of the exoskeleton from the end-user’s point of view, and increase patient training time, since manual tuning of control parameters for each participant is time-consuming de Miguel-Fernández et al. (2023) However, it overlooks the need for personalization in promoting meaningful and task-specific practice Basalp et al. (2021) and potential lasting recovery Lora-Millan et al. (2022). Building on this rationale, several methods for gait-pattern personalization have been developed in recent years, e.g., Carvalho et al. (2025); Challa et al. (2022). For instance, mirroring methods have been proposed to replicate the walking pattern from the unaffected to the affected leg. While promising in the control of leg prostheses Vallery et al. (2011); Wu et al. (2022), in stroke patients, this method does not guarantee the presentation of normal patterns, as the unimpaired side might show compensatory movements Lora-Millan et al., 2022 Model-based methods, instead, generate gait patterns using mathematical Kagawa and Uno (2009) or biomechanical models Falisse et al. (2019); Hu et al. (2022), incorporating parameters like joint angles, muscle forces, and body dynamics. A major drawback of these approaches is their reliance on complex formulations and assumptions about body dynamics and muscle activation that may not fully capture the complexity of pathological human gait. This limitation can be mitigated by learning-based methods, which derive gait patterns directly from data, allowing the representation of nonlinearities and inter-individual differences that are difficult—or even impossible—to encode analytically. This last class of approaches is becoming increasingly popular due to their proven effectiveness in predicting individualized gait patterns Ren et al. (2023); Zou et al. (2021). Recent advances in robot locomotion have demonstrated their growing success for generating natural and adaptive movement patterns Mirza and Singh (2025), at the cost of heavily depending on large, high-quality training datasets Géron (2019).

However, conclusive evidence on the benefits of personalized over generic gait patterns in terms of rehabilitation outcomes remains limited Guo et al. (2022). Particularly noteworthy is the current lack of empirical evidence of the superiority of personalized patterns on users’ subjective experience in terms of comfort, naturalness, and enjoyment, as these factors influence motivation Wulf and Lewthwaite (2016), and participation Yan and Jia (2022). Driven by these challenges, we ran a pilot study to compare unimpaired participants’ subjective perception in response to walking in exoskeleton-enforced personalized and non-personalized gait patterns. We assessed participants’ perception in terms of *enjoyment*, as it is a fundamental element of motivation Center For Self-Determination Theory, 2023, a driver of motor relearning Wulf and Lewthwaite (2016) and therapy adherence Belo Fernandes et al. (2024); *comfort*, since physical strain during exoskeleton use can limit user’s acceptance Wang et al. (2018); and *naturalness*, because more natural movements might favor motor relearning Levac et al. (2019).

We enforced the different gait patterns using an in-house modified Lokomat® exoskeleton. The treadmill-based exoskeleton can support the already integrated knee flexion/extension in the commercial version, as well as newly actuated hip flexion/extension and ab-/adduction together with full translational and rotational pelvis movements, with actuated pelvis lateral movements. To the best of our knowledge, the complete exoskeleton platform and its validated kinematic model have not been presented previously. In addition to evaluating gait personalization, this study therefore presents both the platform and its model. The personalized gait patterns, i.e., hip, knee, and lateral pelvis desired trajectories, were obtained for each participant using gait pattern prediction models based on walking speed as well as anthropometric and demographic data. The models were trained on an extensive online existing walking database. The trajectories were then enforced using a stiff position-derivative controller. We expected participants to prefer their personalized gait patterns in terms of enjoyment, comfort, and perceived naturalness.

## 2 Methods

### 2.1 The treadmill-based lower-limb exoskeleton

For this work, we used a grounded exoskeleton developed at the Sensory Motor Systems (SMS) Lab at ETH Zurich in collaboration with Hocoma AG, Switzerland (Figure 1). While individual hardware subsystems have been described in prior thesis work and technical publications, the complete exoskeleton platform and its kinematic model have not been reported before and are presented here for the first time. The new exoskeleton is a modified version of the company’s well-known Lokomat®. In particular, the system maintains the original Lokomat®, parts of the body-weight support (BWS) system, the treadmill, and the knee joint flexion/extension ball-screw-driven linear actuation. The system also maintains the in-line force sensor at the knee actuation to measure the human-robot force interactionHocoma (2023).

**FIGURE 1:**
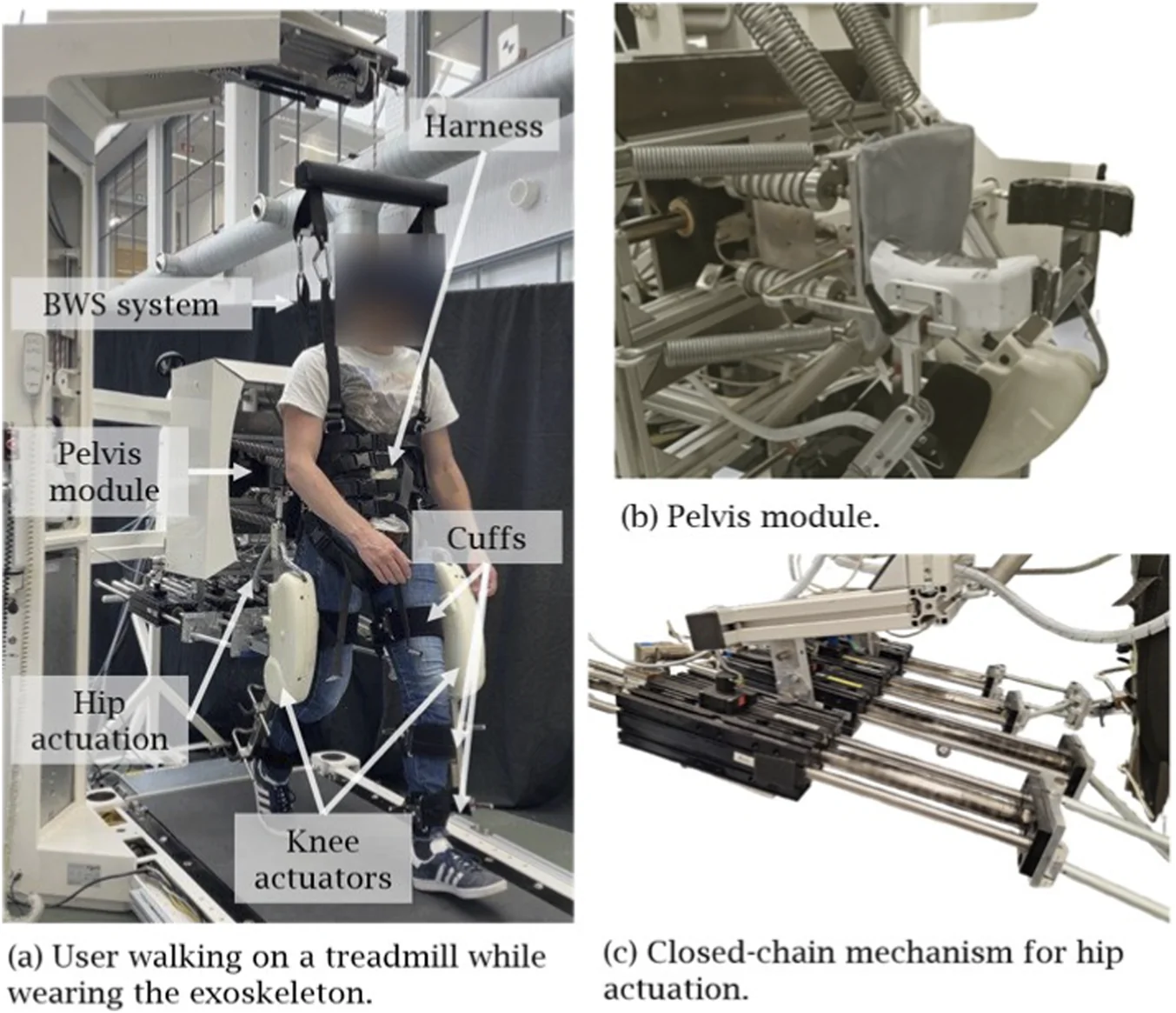
**(a)** The new exoskeleton with a user secured, featuring an active body weight support (BWS) system with actuated lateral movement capability, a 6-DoF series elastic actuation (SEA) pelvis module with additional lateral translation actuator, two active 2-DoF closed-chain hip actuators, ball-screw-driven 1-DoF knee actuators, ankle/shank/thigh cuffs, and a safety harness for fall prevention. **(b)** Detail of the 6-DoF pelvis module. **(c)** Detail of the closed-chain mechanism for hip actuation.

The mechanical innovations of the modified exoskeleton include: i) changes in the hip actuation to allow for hip flexion/extension and ab-/adduction Wyss (2019), ii) a pelvis module that embodies a six degree of freedom (DoF) compliant mechanism that accommodates weight shifting during walking, with one actuated DoF that assists the pelvis lateral movements Wyss et al. (2018), and iii) the original BWS system augmented by one actuated DoF that enables lateral movements to follow the pelvis lateral movements Wyss et al. (2014). These innovations resulted in a human–exoskeleton interface that incorporates twelve degrees of freedom (DoFs): six at the pelvis, two at each hip, and one at each knee. Among these, seven DoFs are actuated: one at the pelvis (coupled with the new actuated BWS lateral mechanism), two at each hip (flexion/extension and ab-/adduction), and one at each knee. The kinematic model of the exoskeleton is described in detail in the Supplementary Materials. Below we summarize the most relevant robot features.

The pelvis module includes a pelvis plate, which is attached to the user’s pelvis and can undergo both translational and rotational motion thanks to a compliant spring-based connection between the fixed back of the pelvis module and the pelvis plate (Figure 1b; see Wyss (2019) for further details). The pelvis module functions as a series elastic actuator (SEA), with one actuated degree of freedom (P01-48x240 motor, NTI AG LinMot, Switzerland), enabling lateral movement relative to a fixed frame. To prevent hindering the natural lateral movement of the pelvis, the BWS is laterally actuated through a lead-screw mechanism parallel to the pelvis’s actuated degree of freedom. Note that the other parts of the BWS remain unchanged with respect to their commercial version, i.e., allow for static and dynamic users’ weight unloading.

Hip actuation is achieved through a closed-chain mechanism Figure 1c driven by two linear actuators per leg (P01-48x240 motors, LinMot, Switzerland). This configuration enables control of both hip flexion/extension and ab-/adduction, while maintaining a rigid joint rotation. Its mechanism converts the independent translational motions of the linear actuators into coordinated movements of the thigh link in the frontal and sagittal planes, as illustrated in Figure 2. In particular, this configuration enforces hip flexion/extension movements when the two linear actuators of a leg move in the same direction Figure 2b, while ab-/adduction movements are produced by moving in opposite directions Figure 2c. By allowing the linear actuators to rotate around the x-axis of the frame located in correspondence to the hip passive joint, the system avoids overconstraining the leg. This mainly parallel actuation configuration offers several benefits over a serial approach, the most notable being a reduction in orthosis inertia, resulting in a more transparent system by design.

**FIGURE 2:**
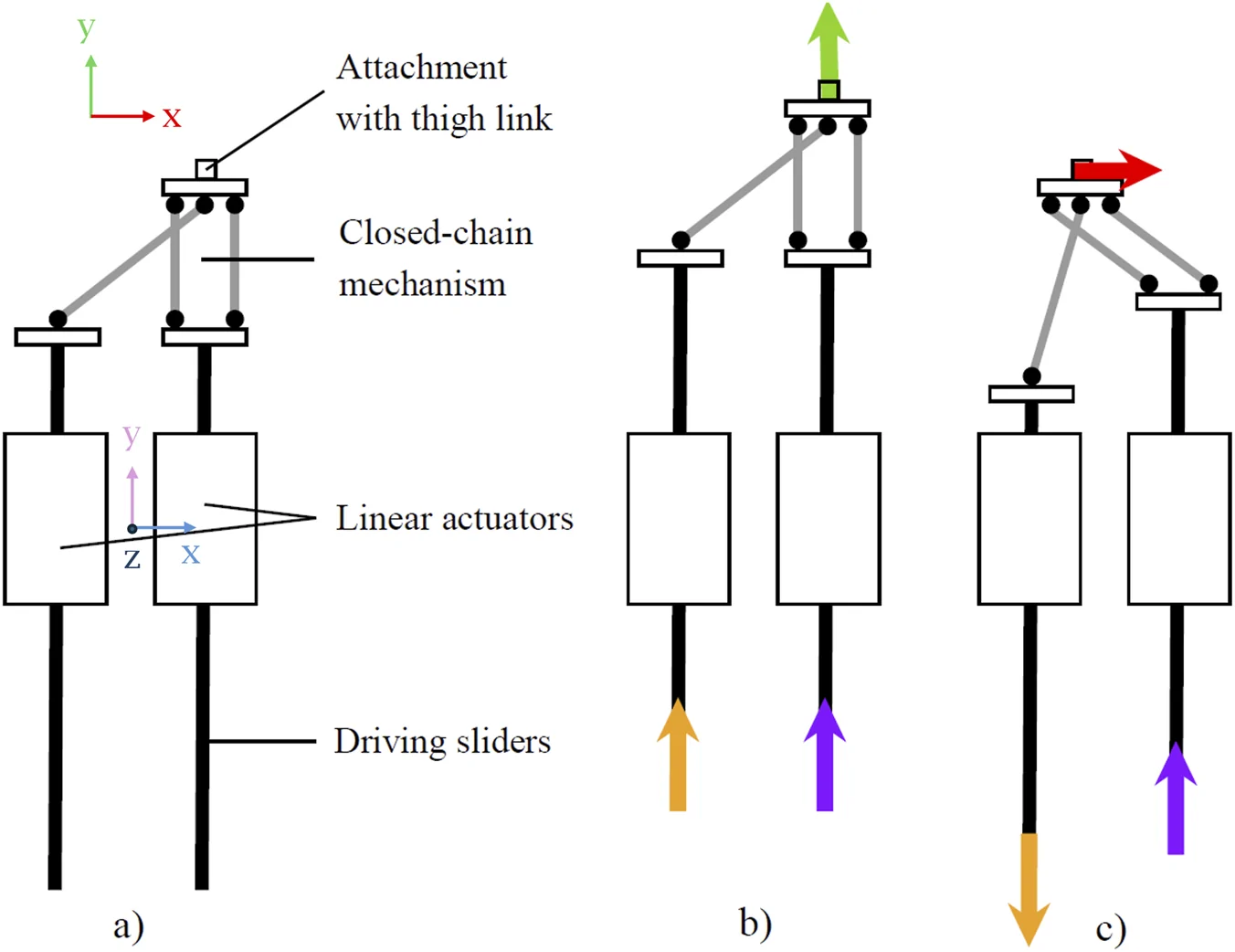
Top view of the right leg’s closed-chain mechanism with the pelvis fixed in rotation, shown in different configurations: **(a)**Neutral, **(b)**Hip flexion/extension motion, and **(c)**Hip ab-/adduction motion. Orange and purple arrows represent the movement of the linear actuators. Red and green arrows represent the resulting motion at the thigh. The blue and pink x-y-z arrows in **(a)**represent the frame located in correspondence to the hip passive joint.

The various actuators have built-in incremental position encoders. The pelvis module includes a Pixart infrared (IR) motion camera (PixArt Imaging Inc., Hsinchu, Taiwan) and a 6-DoF inertial measurement unit (IMU) (MPU9250, InvenSense, San Jose, USA). A Kalman filter is used to combine the IMU and camera data to estimate the position and orientation of the pelvis plate with respect to the fixed back of the pelvis module, see Wyss (2019) for further details.

The exoskeleton is attached to the participant’s lower limbs using the Lokomat® original cuffs placed on the thighs, shanks, and ankles. The participant’s pelvis is secured to the pelvis module between two house-made fixtures positioned on either side, as shown in Figure 1a. Additionally, participants wear a body harness suspended on the BWS. The knee joint is part of the original Lokomat® device.

Since the mechanism of the exoskeleton does not incorporate any sensors to measure the rotations of the hip joint, we developed a closed-form analytical solution for the the direct and inverse mapping between the actuators’ joint coordinates representing the shaft length of the two prismatic actuators of the hip module, and the space of hip joint coordinates, representing the hip flexion/extension, ab-/adduction, and internal/external rotation, respectively. The details of the mapping, together with a detailed description of the kinematic model of the exoskeleton, are presented in the Supplementary Materials.

### 2.2 Gait pattern prediction models

We developed models to predict leg joint and pelvis trajectories based on individual characteristics such as height and age. Our approach builds on the models proposed by Koopman et al. (2014), which use multiple polynomial regression models to predict knee and hip flexion/extension joint trajectories from a person’s height and walking speed. To improve the personalization potential of Koopman’s proposed models, here we also account for the effect of other factors (e.g., age, body weight, and gender), which have been shown to substantially affect joint angles and overall walking patterns Oberg et al. (1994); Hanlon and Anderson (2006); Wu et al. (2018); Ren et al. (2023). We further expanded the model to predict the trajectories of the augmented DoFs of our exoskeleton, namely, the lateral pelvis translation and hip ab-/adduction.

#### 2.2.1 Database

To train and validate our model, we used the publicly available gait database from the Laboratory of Biomechanics and Motor Control at the Federal University of Brazil Fukuchi et al., 2018. This database includes data from 42 volunteers, split into 24 young adults (21 years–37 years) and 18 older adults (50 years–84 years), all without lower-extremity injuries or gait impairments. Data were collected with a marker-based motion-capture system composed of twelve cameras, from participants walking barefoot on a treadmill at eight different speeds, ranging from 40% to 145% of their comfortable, self-selected walking speed. Table 1 summarizes the anthropometric and demographic characteristics of this dataset. Compared to the database used by Koopman *et al.*, which included gait kinematics from fifteen middle-aged participants, this dataset encompasses a broader range of subjects, including individuals in their twenties up to nearly 80 years old.

**TABLE 1:** Descriptive statistics of participants’ anthropometric and demographic data from Fukuchi et al. (2018) used to train our predictive models.

Parameter	Mean	Std	Min	Max
Age (years)	42.64	18.62	21	84
Height (cm)	167.12	11.01	147	192
Mass (kg)	67.76	11.24	44.9	95.4

The dataset includes both raw—e.g., marker coordinates and external forces—and processed data—e.g., knee and hip flexion/extension and hip ab-/adduction joint trajectories for each participant at recorded speed level. The database did not include the processed data regarding the lateral pelvis trajectories, so we derived them from the raw marker trajectories. All joint trajectories, including the newly computed lateral pelvis movement, were represented as time-normalized ensemble averages for each participant at their respective gait speeds.

While the database included treadmill speeds up to 8.02 km h^−1^, our treadmill has a speed limit of 3.2 km h^−1^ Hocoma (2023). Therefore, only a subset of the speeds recorded in the original database was included for training our gait pattern prediction model, namely,.

- Level 1, 40% of self-selected speed: 1.80± 0.231 km h^−1^;
- Level 2, 55% of self-selected speed: 2.46± 0.336 km h^−1^;
- Level 3, 70% of self-selected speed: 3.14± 0.410 km h^−1^.

#### 2.2.2 Prediction models for gait key events

Following Koopman *et al.* approach, we used regression models to predict a sparse set of key points within the joint trajectories, called key events. These events are spaced to capture the trajectory’s overall shape, allowing full personalized gait patterns to be reconstructed through interpolation. From the hip and knee trajectories, six key events each were selected, namely, the start of the joint trajectory (heel contact) and the maximum values in position and velocity during both swing and stance phases Koopman et al. (2014). For the lateral pelvis movement, only four key events were employed, corresponding to the point of maximum and minimum pelvis position and velocity along the trajectory. Each event is defined as a set of four parameters: (i) timing (t) expressed as a percentage of the gait cycle, (ii) angle or displacement (y) , (iii) (angular) velocity $\left(\dot{y}\right)$ , and (iv) acceleration $\left(\ddot{y}\right)$ ; see Figure 3 for exemplary right leg trajectories with key events of a single participant at the three walking speed levels.

**FIGURE 3:**
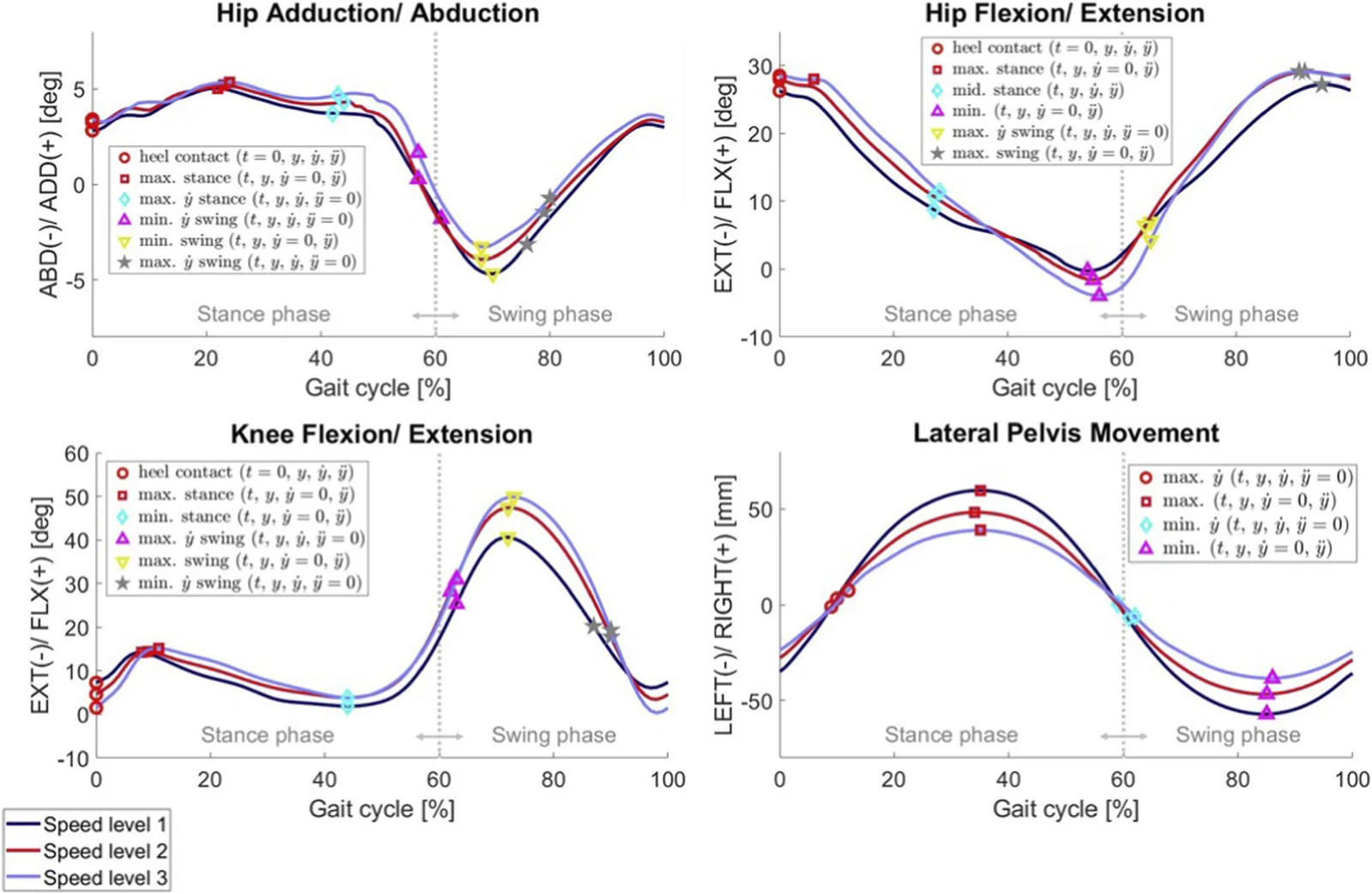
Exemplary right leg trajectories of a participant are displayed at walking speed levels 1, 2, and 3, together with their extracted key events. These key events are the same as in Koopman’s study Koopman et al. (2014), except for lateral pelvis movement (not present in their study), for which extreme position and velocity values were chosen. The key events for the left and right joint trajectories were extracted separately. By default, heel strike timing is set to zero (% of the gait cycle), as are the minimum and maximum values for joint position and velocity. These constraints are detailed in the key event legend.

We trained regression models to predict each of these four parameters, which were then used to reconstruct the final personalized trajectories. We employed the following initial main regression equation, inspired by Koopman et al. (2014):

$$Y=\beta_{c}+\beta_{v}v+\beta_{v^{2}}v^{2}+\beta_{h}h+\beta_{w}w+\beta_{a}a+\beta_{s}s,$$ (1)

where v represents walking speed, h body height, w body weight, a age, and s gender, encoded as a numerical value where female is −1 and male is 1. The output Y represents the predicted key event parameter, i.e., time (t) , position (θ) , velocity $\left(\dot{y}\right)$ , or acceleration $\left(\ddot{y}\right)$ . The inclusion of both linear and quadratic walking-speed terms reflects the nonlinear relationship between gait speed and lower-limb kinematics and provides flexibility to capture nonlinear variations Lelas et al. (2003).

We fit one regression model per parameter for all the key events, leading to 24 regression models for each hip ab-/adduction, hip flexion/extension, and knee flexion/extension trajectory (i.e., four parameters per each of the six key events per trajectory), and 16 models for the lateral pelvis movement (with only four key events). To prevent overfitting and improve interpretability, stepwise regression was conducted to evaluate the significance of the predictor variables in Equation 1. Only variables with significant effects (p<0.01) were retained, following the Koopman *et al.* approach Koopman et al. (2014). Then, robust regressions with a *bisquare* weighting function were employed to estimate the final regression coefficients $\left(\beta_{x}\right)$ . In this way, different models could rely on different predictors (see the Supplementary Materials for the complete regression equations derived for the different joint trajectories).

An additional regression model, again reduced through stepwise regression to identify significant effects, was derived from Equation 1 to predict the gait cycle time, resulting in:

$$T_{\mathrm{pre}}=2.7662-0.7458v+0.0903v^{2}-0.0037a,$$ (2)

where the gait cycle time $\left(T_{\mathrm{pre}}\right)$ depends on the walking speed (v) and age (a) .

#### 2.2.3 Generation and evaluation of personalized gait patterns based on predicted key events

From the predicted four parameters of each gait key event (i.e., t , y , $\dot{y}$ , or $\ddot{y}$ ), we then generated personalized continuous joint kinematic trajectories. We employed the 5th-order piece-wise quintic splines interpolation method, as proposed by Koopman et al. (2014).

The accuracy of the predicted trajectories was evaluated using the data from the publicly available database Fukuchi et al. (2018). We computed the root mean square error (RMSE) against the real database trajectories, using the leave-one-out cross-validation method. The results were then averaged across all participants and over both left and right joint trajectories.

#### 2.2.4 Generation and evaluation of the *standard gait pattern*

Alongside the *Personalized Gait Pattern*, we used the same database to define a *Standard Gait Pattern*, meant to provide a standard pattern ideally suitable for all participants. This standard pattern was obtained by averaging the gait patterns of the three included walking speed levels across all participants in the dataset. We globally assumed symmetrical gaits, so the trajectories of both the left and right legs were combined. Note that this average was taken over the entire trajectory, rather than averaging over the key events.

Because this was conceived as a standard gait pattern for all scenarios, we, therefore, had to adjust the trajectories by estimating the gait cycle time $\left(T_{\mathrm{stand}}\right)$ to account for different desired gait speeds. Therefore, a separate regression model (Equation 3), reduced following the same steps described for Equation 2, was employed to estimate the gait cycle time also for the *Standard* gait pattern:

$$T_{\mathrm{stand}}=1.8993-0.6909v+0.0789v^{2}+0.3928h.$$ (3)

We calculated the RMSEs between the standard and actual gait patterns from the database as a means to compare their accuracy with that from the *Personalized* gait pattern.

### 2.3 Pilot user evaluation of the personalized and *standard gait patterns*

#### 2.3.1 Participants

Ten unimpaired participants participated in the pilot study, equally divided into females and males. Their ages ranged from 2 years to 27 years, with a mean age of 25 years, average height of 1.76± 0.089 m, and weight of 69.25± 12.79 kg. None reported neurological or orthopaedic disorders. The study was approved by the TU Delft Ethics Committee (HREC, Application 25-08-2023) and was conducted in accordance with the Declaration of Helsinki. Prior to the experiment, all participants read and signed an informed consent form for data and image collection and publication in an anonymized form.

#### 2.3.2 Experimental protocol

Participants underwent three different test conditions (trials), of two minutes each, with the exoskeleton guiding their lower limbs along three distinct gait patterns: (i) the *Personalized* gait pattern, (ii) the *Standard* gait pattern, and (iii) a *Random* gait pattern. The order of the gait pattern condition trials was pseudo-randomized across participants, i.e., each of the six possible condition sequences was used once (following a Latin square), and then four more sequences were randomly selected from these six. The trial duration was chosen to balance the time needed to experience the pattern with the risk of fatigue.

The first two gait patterns were introduced in detail in Section.2.2. For the *Random* gait pattern condition, each participant was assigned a gait pattern randomly selected from the recorded patterns from Fukuchi’s database Fukuchi et al. (2018). The sampling was restricted to patterns that fell within the previously selected subset of speeds. The inclusion of this random pattern served as a baseline to observe the effects of non-tailored but still physiologically plausible assistance, resembling current clinical practice where pre-recorded gait patterns are used as input to trajectory-based controllers.

Participants were instructed to remain passive to the movement of the exoskeleton and received no indication about the differences among gait pattern conditions. For all conditions, the walking speed was set to 1.8 km h^−1^, reflecting the average speed of the lowest walking speed level in the database. Only for two participants, who explicitly requested to increase the speed to walk more comfortably, it was set to 2.0 km h^−1^, and patterns were adjusted accordingly. While participants were wearing the harness for safety, the BWS system remained slack, such that participants did not receive any weight compensation.

We operated the exoskeleton in Proportional-Derivative (PD) control, with the computed gait patterns as reference trajectories. The controller was intentionally rigid (for the hip and pelvis PD controller: Kp= 5.0 A mm^−1^ and Kd= 7.5 A/(m/s), for the knee PD controller: Kp= 1.0 A mm^−1^ and Kd= 0.1 A/(m/s)), allowing minimal compliance to enhance the user’s sensitivity to variations in gait patterns. We also aimed to achieve stiff connection points between the participants’ limbs and the robotic device at the pelvis, thighs, and shanks to make trajectory differences more perceptible to the participants.

After signing the informed consent form, participants wore the exoskeleton, which was adjusted to each of their body dimensions, including the width of the pelvis, the frontal position of the hip joint, thigh length, and shank length. The kinematic model of the exoskeleton was adjusted according to these dimensions. The reference actuator position trajectories were then generated using the exoskeleton model (see Supplementary Materials) and based on hip ab-/adduction, hip flexion/extension, and pelvis lateral motion calculated patterns. Once the exoskeleton was fit and the exoskeleton kinematic model adjusted, participants underwent a familiarization phase lasting around two minutes before testing the three different gait pattern conditions. During this initial phase, participants walked with the exoskeleton in a ‘transparent mode’ Marchal-Crespo and Reinkensmeyer (2009), i.e., they could move as freely as possible while getting used to walking with the exoskeleton. Emergency stops were provided to both the participant and the experimenter.

#### 2.3.3 Data acquisition and processing

After each condition, participants filled in a questionnaire to evaluate their perceptions of the different gait pattern conditions with regards to.

- Interest/Enjoyment: Measured with four items from the Intrinsic Motivation Inventory (IMI) Center For Self-Determination Theory, 2023, with scores averaged per IMI guidelines.
- Comfort: Assessed through seven self-designed questions to capture various aspects of comfort experienced during exoskeleton use.
- Naturalness: Evaluated with four self-designed questions about how participants perceived the movements of the exoskeleton, analyzed individually.
- Passiveness: Evaluated through four self-designed items to determine whether participants remained passive using the exoskeleton, with scores averaged to assess overall passiveness.

The full list of questions/items employed can be found in the Supplementary Table S6. Participants filled in the questionnaires following a 7-point Likert scale, where, according to the question: (1) Very unnatural/uncomfortable/dissimilar - (7) Very natural/comfortable/similar or (1) Not true at all - (7) Very true.

At the end of the experiment, participants also ranked—from (1) Favorite to (3) Least favorite—the three gait patterns in terms of overall experience, comfort, and naturalness. They also rated their confidence in these rankings, from (1) Not confident to (10) Very confident. Finally, we included open-ended questions (reported in the Supplementary Materials) to capture additional insights and comments.

Besides the participants’ subjective experience, joint positions and forces measured by the original knee joint force sensor were recorded throughout the experiment at a sampling frequency of 100 Hz. To determine whether walking with different gait patterns affected human-robot interaction, the Mean Absolute Force was calculated per condition and leg as the average magnitude of the force exerted at the right/left knee joints. To assess the exoskeleton’s ability to reproduce the commanded trajectories, tracking error was quantified as the RMSE between the commanded and measured actuator trajectories. Tracking RMSE was computed for each participant and actuator over the gait cycle and subsequently averaged across participants. Then, to quantify the differences between the enforced gait patterns, we computed the RMSE between each pair of commanded trajectories (Personalized vs. Standard, Personalized vs. Random, and Standard vs. Random) for each participant and actuator. The resulting RMSE values were then averaged across participants (Supplementary Table S6). To contextualize the magnitude of these inter-pattern differences, they were compared with the corresponding exoskeleton tracking errors.

#### 2.3.4 Data analysis

We evaluated the effect of the different gait pattern conditions on the subjective and objective performance variables using a linear mixed model (LMM) of the form:

$$Var=Pattern+TrialOrder+\left(1|Participant\right).$$ (4)

In Equation 4 Var refers to any of the measured performance metrics (e.g., Comfort). The independent variable Pattern refers to the pattern condition (i.e., *Personalized*, *Standard*, or *Random*). The *Standard* condition was chosen as the base condition, being equivalent for all the participants. To control for potential effects related to the sequence in which conditions were presented, we also included the TrialOrder as an independent variable in the model. This ordinal variable represents the position of each pattern condition within the experimental sequence for each participant (i.e., First, Second, or Third). We also included Participant as a random factor. We fit the model using the lmer function from the lme4 package in R.

Additionally, ranking outcomes were analyzed using a Friedman test, comparing across experimental conditions and trial order. After identifying significant differences with the Friedman test, a post-hoc Nemenyi test was conducted to explore pairwise comparisons between conditions. This analysis was performed using the frdAllPairsNemenyiTest function from the PMCMRplus package in R. We set all significance levels to p<0.05 and corrected for multiple comparisons with Bonferroni correction.

## 3 Results

### 3.1 Accuracy evaluation of the personalized and Standard gait patterns

Results from the leave-one-out cross-validation method used to assess the RMSEs between the *Personalized*/*Standard* and actual gait patterns are reported in Table 2. We did not observe large differences in accuracy between the *Personalized* and *Standard* gait patterns. For the hip joint trajectories, the *Standard* pattern’s RMSE values were slightly lower compared to the *Personalized* ones. Conversely, for the knee and pelvis joint trajectories, the *Personalized* reconstructed trajectories had slightly lower RMSE than the *Standard* trajectories. Thus, neither model consistently outperformed the other across all evaluated joints.

**TABLE 2:** RMSE results from the leave-one-out cross-validation method used to assess the accuracy of the *Personalized*/*Standard* generated patterns against actual patterns from the database.

Joint	RMSE (actual–Personalized)	RMSE (actual–Standard)
Hip abduction/adduction (°)	2.906	2.772
Hip flexion/extension (°)	7.573	6.953
Knee flexion/extension (°)	5.809	6.385
Lateral pelvis movement (mm)	6.321	6.779

### 3.2 User experience

Results from the LMM for the user experience analysis, together with the average scores assigned per gait pattern condition are reported in the Supplementary Table S8, Supplementary Table S6. Here, we comment on relevant results.

In terms of *Comfort*, mean scores were 3.34 for the *Personalized* condition, 4.09 for the *Standard* condition, and 3.69 over 7 for the *Random* condition. We found that the overall comfort significantly improved in the third trial with respect to the first one (β=1.43,t=2.94,p=0.01) . No significant differences were perceived among conditions. When looking into the individual *Comfort* sub-questions, we found a significant effect of the gait pattern condition on the perceived *Physical strain*. In particular, participants reported higher *Physical strain* when walking with the *Personalized* gait pattern vs. the *Standard* pattern condition (β=1.07,t=2.86,p=0.011) . Interestingly, *Physical strain* seemed to decrease with trials, albeit non-significantly (β=−0.26,t=−0.70,p>0.05) .

Regarding *Naturalness*, with mean scores of 2.85, 3.56, and 3.39 over 7 for the *Personalized*, *Standard*, and *Random* conditions, respectively, we also found a main effect of the condition order. In particular, the third trial was perceived significantly as more natural than the first trial (β=1.35,t=2.79,p=0.013) , and as more similar to the participants’ own way of walking (β=1.85,t=3.59,p=0.002) . Questions related to the *Smoothness of movements* and whether the limbs were pushed beyond their natural range did not show significant differences across conditions or condition order.

The models for both the *Interest/Enjoyment* (IMI) and *Passiveness* metrics revealed no significant differences, either in terms of gait pattern or condition order. The *Personalized* pattern got the highest enjoyment score, but differences with the other patterns were not significant (β=0.20,t=1.35,p>0.05) .

When analyzing self-reported condition rankings, we did not find significant differences across the gait patterns, including overall preference, comfort, and naturalness (see Table 3).

**TABLE 3:** Friedman test results for participants’ rankings. When the test was significant, we performed pairwise comparisons across patterns - Standard (S), Personalized (P), and Random (R) - or across experimental trials – 1st: T1, 2nd: T2, 3rd: T3.

Comparison groups	Ranking	Friedman test			Post-hoc comparison p -values		
		$\chi^{2}$	df	p -value	S vs. P	S vs. R	P vs. R
Conditions (patterns)	Overall preferred pattern	4.1	2	0.122	-	-	-
	Most comfortable pattern	3.2	2	0.202	-	-	-
	Most natural pattern	2.6	2	0.272	-	-	-
		$\chi^{2}$	df	p -value	T1 vs. T2	T2 vs. T3	T1 vs. T3
Trial order	Overall preferred pattern	5.6	2	0.061	-	-	-
	Most comfortable pattern	6.2	2	0.045*	0.261	0.644	0.037*
	Most natural pattern	6.2	2	0.040*	0.261	0.644	0.037*

* (p<0.05) .

However, we found that the order of the trials seemed to play a role in the participants’ rankings. In particular, we found that the third trial was generally perceived as more comfortable (p=0.037) and more natural (p=0.037) compared to the first trial. This comes with a general high confidence in participants’ rankings, scoring 8.2±0.92 regarding overall preference, 8.3±1.06 regarding the most comfortable gait pattern, and 7.6±1.51 regarding the most natural gait pattern, on a scale from 1 (not confident at all) to 10 (very confident). For completeness, the responses to the open-ended questions are included in the Supplementary Materials.

### 3.3 Human-robot interaction forces and kinematics

The results for the LMM analysis of the mean absolute force at both knee actuators are reported in Table 4. We found a significantly higher force when participants were enforced to follow the *Random* gait pattern when compared to the *Standard* gait patterns (Right leg: β=68.7,t=3.6,p=0.002 ; Left leg: β=84.5,t=4.1,p=0.001 ). The difference between the *Personalized* and *Standard* gait patterns only approached significance on the left leg (Right leg: β=22.2,t=1.2,p=0.257 ; Left leg: β=42.1,t=2.0,p=0.061 ), with higher forces for the *Personalized* trajectories. We did not find a significant effect of the trial order on the interaction forces.

**TABLE 4:** inear mixed-model results for mean absolute knee actuator force (N).

Variable	Right knee				Left knee			
	β	SE	t	p	β	SE	t	p
Intercept	153.4	20.5	7.5	8.22E-08***	140.0	24.1	5.8	5.78E-06***
Personalized pattern	22.2	18.9	1.2	0.257	42.1	20.9	2.0	0.061
Random pattern	68.7	18.9	3.6	0.002**	84.5	20.9	4.1	0.001**
2nd trial	−19.7	18.9	−1.0	0.313	−17.7	20.9	−0.8	0.409
3rd trial	−25.6	18.9	−1.4	0.193	−19.2	20.9	−0.9	0.372

** p<0.01 , *** p<0.001 .

Results of our tracking accuracy analysis showed that tracking errors remained low across all actuated joints (maximum 8.48 mm for prismatic actuators and 3.33 ◦ for revolute actuators), confirming that the stiff PD controller accurately reproduced the desired gait patterns (Supplementary Table S5). When compared with the tracking errors, the magnitude of the differences among Standard, Random, and Personalized patterns (Supplementary Table S6) showed that the imposed trajectory differences were substantially larger than the device tracking errors. For instance, for the knee joints, the average inter-pattern differences were approximately 24–26 ° , whereas the corresponding tracking errors were only 2.9–3.1 ° . Similarly, for the pelvis and hip-related prismatic actuators, trajectory differences exceeded tracking errors by approximately one order of magnitude.

## 4 Discussion

### 4.1 A novel exoskeleton for enhanced degrees of freedom

We presented, for the first time in the peer-reviewed literature, a novel exoskeleton and accompanying validated kinematic model based on a modified Lokomat®. The device includes the standard configuration with augmented hip ab-/adduction and full pelvis translation and rotation (with actuated lateral translation), allowing for more realistic gait training. We also implemented a gait personalization algorithm, based on a comprehensive walking database, that can predict individualized gait patterns based on individuals’ walking speed, anthropometric, and demographic characteristics. Whereas the commercial version of the Lokomat® relies on predefined physiological profiles with heuristic clinician-adjustable parameters Marchal-Crespo et al. (2019); Marchal-Crespo and Riener, 2022, our framework generates population-informed trajectories from individual user characteristics and may therefore better represent physiological inter-individual variability without the need for tedious empirical adjustments. Yet, this potential advantage was not directly evaluated here. We evaluated the potential of our system’s personalized trajectories against non-personalized ones from a public gait dataset in a pilot user experience study with ten unimpaired participants.

The results of the overall user experience indicate that both comfort and naturalness could be improved, regardless of the enforced gait pattern. Participants’ feedback indicated that there was significant discomfort associated with the device, particularly at the ankle cuff. Moreover, participants commonly mentioned they were experiencing excessive and unnatural lateral pelvis movement. This could be due to the compliance of the pelvis module itself. Instructing participants to remain passive—that they seemed to achieve based on the results from the *Passiveness* questionnaire—may have caused them to let the exoskeleton sway their upper bodies side to side, potentially increasing the sensation of excessive lateral movement. The relatively slow walking speed enforced by the exoskeleton might have also contributed to the general unnatural feeling, as the average comfortable speed in healthy adults is around 5.0 km h^−1^ Baroudi et al. (2022). Nevertheless, as discussed in more detail below, the overall experience improved significantly over time, and therefore, results regarding overall experience with the exoskeleton within this pilot study should be taken with care.

### 4.2 Personalization did not significantly influence user experience

Contrary to our expectations, we did not find statistically significant differences in the reported user experience—namely *Interest/Enjoyment*, overall *Comfort*, and *Naturalness*—between the *Personalized*, *Standard*, and *Random* gait patterns.

We only found a small, albeit significant, higher perceived physical strain in the *Personalized* vs. *Standard* pattern. This increased physical strain could be due to the model predicting the trajectories of different joints separately, without taking into account their inter-joint coupling during walking. This might lead to a reduction in enforced inter-joint coordination. Nevertheless, this perceived physical strain was not reflected in significant differences in human-robot interaction forces between these two specific conditions, as only a non-significant increase in force at the left knee was observed in the *Personalized* vs. *Standard* gait pattern, nor did participants report a lack of coordination during the open questions interview. The only significant force differences were observed between the *Random* and *Standard* patterns, with larger interaction forces associated with the former pattern in both legs. Nevertheless, this increase was not accompanied by a statistically significant reduction in perceived knee comfort ( β=−0.55 , p=0.320 ), although the effect was in the direction of lower comfort. This suggests that subjective comfort may depend not only on the magnitude of forces at a single location but also on the overall physical interaction with the exoskeleton. Since interaction forces were measured exclusively at the knee interface, the present analysis could not assess force redistribution across other contact points of the device. Furthermore, participants likely evaluated comfort within the broader context of the overall walking experience. For healthy individuals, this global perception of robot-assisted walking may have been more salient than localized differences in knee interaction forces, potentially explaining the discrepancy between objective force measurements and subjective comfort ratings.

While healthy gait is, overall, unique and repeatable, the natural fluctuations in human motor control could explain why preference was similar across the three profiles. Healthy gait exhibits non-negligible stride-to-stride variability Moreira et al. (2021); Hausdorff (2007) and is robust to changes in speed, environment, and perturbations ? Kinematic variability also increases at slower than preferred walking speeds, such as the speeds experienced in our setup Terrier and Schutz (2003); Yu et al. (2009). This implies that one person’s average gait trajectory is not rigidly distinct from another’s but is instead likely to fall within the broader distribution of kinematic patterns expressed by other healthy young adults. Such overlap reflects the redundancy and flexibility of the neuromotor system, which allows different movement strategies to achieve stable locomotion Stergiou and Decker (2011). Thus, the result that participant preference did not vary between the personalized and non-personalized conditions may reflect this balance between individuality and robustness. We may then expect different results in impaired populations, in which gait variability is elevated relative to healthy young adults Moon et al. (2016). Automatic personalization of therapy—such as through human-in-the-loop robotic tuning Slade et al. (2024)—is important to address the diverse and evolving needs of patients throughout rehabilitation.

Additionally, systemic differences between the imposed and real-world gaits may overwhelm differences between the three conditions. Exoskeleton locomotion differs qualitatively from normal, unconstrained gait Swank et al. (2019), so users may not consciously perceive the nuances between patterns. For example, people without prior experience with exoskeletons prefer lower levels of assistance Swank et al. (2019); Ingraham et al. (2022), suggesting that users may be more affected by the intrusiveness of the exoskeleton guidance. In our study, trajectories were enforced using a rigid position-derivative controller, selected intentionally to amplify the perceptibility of the trajectories for participants, but this might have contributed to participants’ discomfort. While rigid guidance was meant to emphasize differences, it also reduced compliance at the joints. Overall, this might have led to a general sense of unnaturalness that overshadowed the subtler distinction between patterns. The trajectories were also separately determined for each degree of freedom. This decoupling of joint kinematics may degrade the inter-joint coordination necessary for stable gait Chiu and Chou (2013), which could explain the lower scores, especially for the personalized pattern. We, therefore, cannot decouple the effects of the pattern definition from any possible benefits of personalization.

### 4.3 User adaptation to the experimental setup seems to have a stronger effect on user experience than personalization

It is important to note that while we did not capture significant differences in the user experience between gait pattern conditions, we still found differences between trials. The user experience seemed to improve in terms of overall comfort, naturalness, and smoothness of the exoskeleton’s movements as the experiment advanced, reaching significant differences between the last and first conditions, regardless of the gait pattern enforced. These differences suggest that participants probably adapted to the system over time, leading to an enhanced walking experience. This is supported by the overall higher rating of the last trial as the most comfortable and natural pattern compared to the first one, while no differences in rating were found between gait patterns.

These ratings raise questions about whether participants actually felt differences between gait patterns, despite their overall confidence in their responses. The trajectory analysis confirmed that the generated patterns were quantitatively different, while the cross-validation results showed comparable overall prediction accuracy for the *Personalized* and *Standard* models with respect to the actual database patterns (Table 2). Therefore, the null perceptual findings may be linked to other factors, including, for example, the compliance of the human-robot interfaces. The Velcro® straps and soft leg tissue, specifically, may have attenuated the perception of these differences.

Exposure is known to be an important factor underlying human-robot performance Ingraham et al. (2022); Poggensee and Collins (2021); Tankink et al. (2025). While some studies indicate that significantly more time is required to adapt to exoskeleton assistance Gordon and Ferris (2007); Poggensee and Collins (2021), these results suggest that people begin to enjoy the device more within only a few minutes. The paradigm presented in this study importantly differs from those other exoskeletons—as the goal was to fully guide the user rather than to partially augment existing capabilities—although our results still highlight the need to consider user adaptation when designing human-robot interaction protocols. Adaptation occurs over several different timescales, with kinematic adaptation occurring fairly quickly Gordon and Ferris (2007). It was for this reason that we included a first familiarization period of around two minutes. We then chose 2-min trials for each condition, balancing the time required to form an opinion of the pattern at hand while reducing the potential for fatigue that could occur had the experiment been too long. Nevertheless, the observed order effect suggests that participants were still adapting to the robotic assistance, potentially masking the influence of trajectory personalization. A longer familiarization phase, combined with adequate rest before the first condition, might therefore have reduced this effect.

### 4.4 Study limitations

Our work presents limitations related to the employed prediction model. First, the dataset utilized in this study, from Fukuchi et al., 2018, may not fully represent the anthropometric variability observed in broader populations (e.g., differences in height Data Pandas (2026)), which could affect the generalizability of the results. Second, even if the prediction model incorporates anthropometric, demographic, and speed variables, these factors do not fully capture subject-specific gait characteristics. Individuals with similar predictor values may still exhibit different gait patterns due to differences in neuromuscular, physical, and behavioral factors. Moreover, the narrow ranges of both participant age (23–27 years) and training speeds limited the effective contribution of these predictors to the generated trajectories. Even if the low-speed range used to train our models covers the speed region where gait variability is reported to be greatest (<3.7 km/h) Murakami and Otaka (2017), generalization to higher walking speeds remains uncertain. Furthermore, the personalized trajectories were reconstructed from predicted key events using quintic splines, whereas the standard trajectories were obtained by averaging full gait cycles. This methodological asymmetry may have contributed to differences unrelated to personalization itself. In light of all these elements, the proposed framework should be regarded as a population-informed personalization approach rather than a fully individualized gait reconstruction method, driven predominantly by anthropometric characteristics and low-speed gait behavior.

Several limitations relate to the study protocol. Although 2-min trials represented a reasonable compromise, a longer familiarization period may have better accounted for adaptation to the experimental setup. The study included only young (between 20 and 30 years old) unimpaired participants. Therefore, conclusions represent evidence of feasibility and user perception among unimpaired individuals rather than of clinical efficacy in patient populations. Compared to locomotor patterns in healthy young adults, individuals with neurological disorders often present larger gait variability and compensatory strategies as a result of altered motor control Moon et al. (2016). They may therefore benefit differently from personalized trajectories and may prioritize aspects such as stability, confidence, or fatigue reduction over perceived naturalness. The small sample size, the treatment of ordinal Likert data as continuous, and the presence of an order effect might also limit the robustness of the statistical analysis.

Participants’ feedback suggested that the reference gait patterns were perceived as unnatural and uncomfortable. Such perceptions are likely influenced not only by the patterns themselves but also by the mechanical structure and control strategy of the robotic system. Factors that may have masked personalization effects include the specific cuff design, the intrusiveness of full trajectory guidance, and the independent prediction of joint trajectories. Uncontrolled factors, such as personal footwear—standardized in the reference dataset Fukuchi et al. (2018)—may also have influenced perception.

Future work should experimentally investigate these factors by testing alternative cuff designs, interface stiffness, comparing fully guided and assistive/impedance-based control modes with trajectory-free frameworks such as energy-shaping controllers Zhang et al. (2023), and implementing coupled prediction models that explicitly encode inter-joint dependencies. Other points for improvement include expanding gait databases to include more diverse populations and walking speeds, exploring alternative prediction models or online adaptation methods to capture individual characteristics beyond anthropometric descriptors, and examining perceptions in individuals with stroke. Ultimately, trajectory generation should not only aim for accurate prediction but also allow refinement (e.g., step length or width adjustments) based on individual user feedback. Engaging directly with the final users to gather and analyze their feedback on gait adjustments should be a central focus in future research.

## 5 Conclusion

In this within-subject pilot study, we explored user perceptions of personalized versus standard and random gait patterns in a sample of healthy participants. We employed an exoskeleton capable of multi-planar motion assistance, focusing on enjoyment, comfort, and naturalness. We developed and evaluated a comprehensive kinematic model for the exoskeleton control and regression-based predictive models generating personalized hip, knee, and pelvis trajectories from anthropometric, demographic, and walking speed data. Subjective evaluations revealed minimal differences between gait pattern conditions. We found, however, that participants generally rated the gait pattern experienced last as more comfortable and natural compared to the first one, regardless of the gait pattern enforced, suggesting adaptation to the experimental setup. These findings indicate that, in a small representation of unimpaired users, personalization of gait kinematics has a reduced short-term impact compared to adaptation to the robotic system and overall system ergonomics. Future work should validate the robotic and personalization algorithm framework in clinical populations, where personalization may play a more critical role.

## Data Availability

The datasets generated and then analyzed for this study can be found on Zenodo at 10.5281/zen549 odo.17985946.
