# Supplementary material for: The impact of gait pattern personalization on the perception of rigid robotic guidance: a pilot user experience evaluation
Source: Front Robot AI. 2026 Oct 30;13:1937649. doi: 10.3389/frobt.2026.1937649 (PMC13759802; doi:10.3389/frobt.2026.1937649)
Supplement: Supplementary file 1 [file DataSheet1.pdf]

Supplementary Material

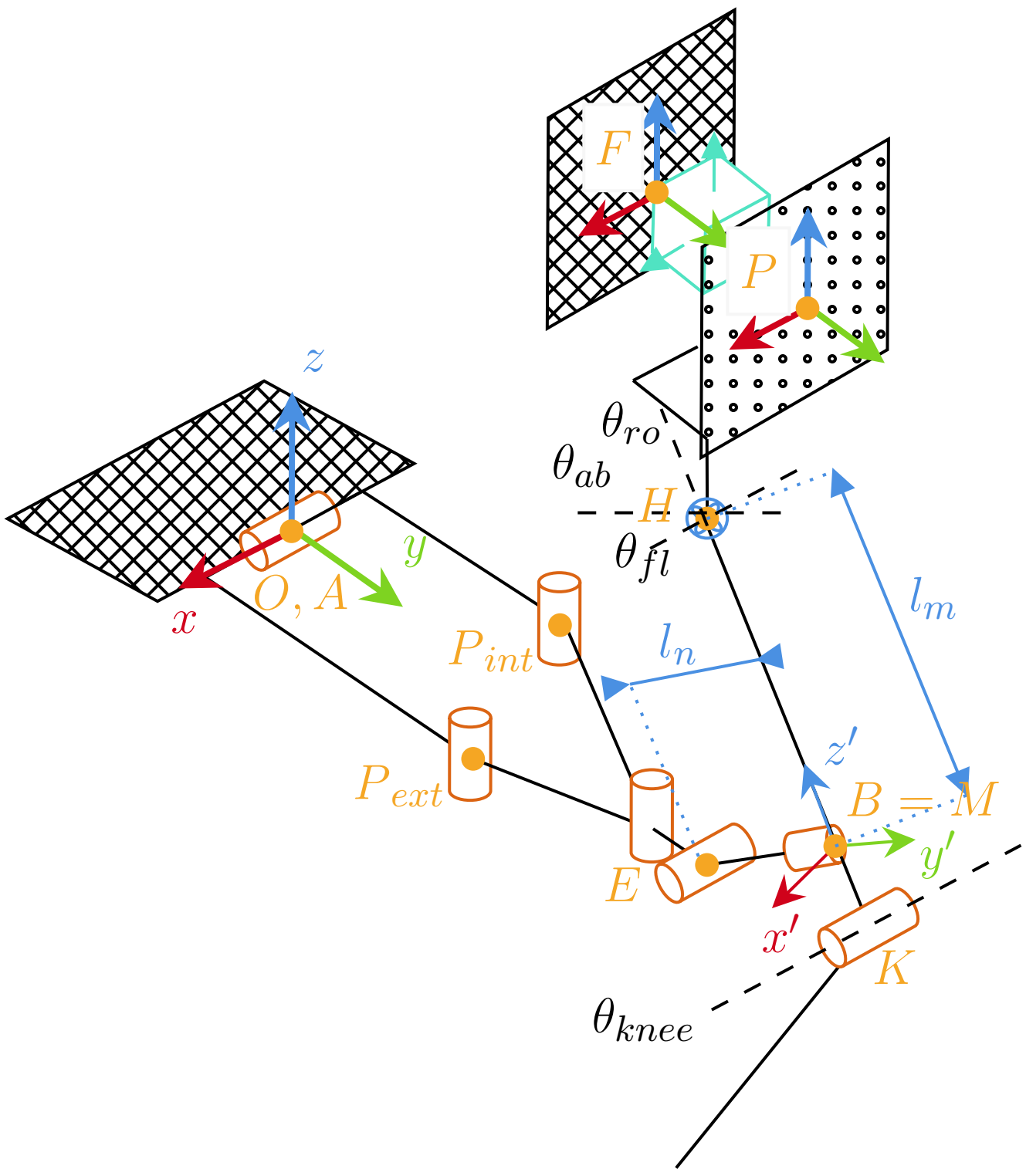

(a) Whole schematic.

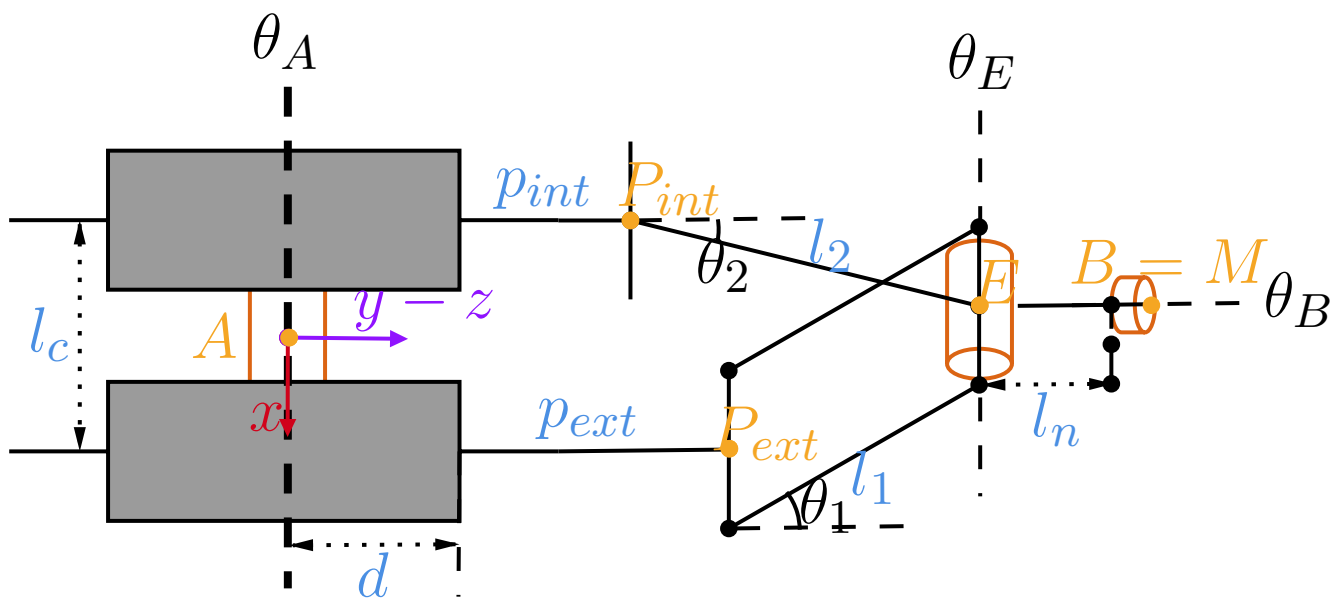

(b) Hip actuators in actuator plane.

Figure S1: (a) Schematic of right leg kinematics where O is the origin of the global frame, A the origin of rotation axis of the hip actuators, P_{int} and P_{ext} are foreheads of the prismatic shafts, E end-effector point of the parallel mechanism, B head point of the back shaft, M the projected point of B on the thigh link, H the hip joint, P the pelvis plate and F the fixed pelvis frame, and K the knee joint. l_m and l_n are fixed segment lengths, with l_n being the distance between the two non-actuated perpendicular revolute joints centered in E and B, and l_m the thigh link segment length (which can be modified based on the user's thigh length). (b) Top view of the hip actuators with parallel mechanism in the actuator plane; l_c is the distance between the two parallel motors' linear shafts, d is half the length of the linear actuators, and P_{ext} and P_{int} represent how far the motor shafts travel. θ_A, θ_B, and θ_E are unknowns.

1 KINEMATIC MODEL

1.1 Global description

Fig. S1.a represents the full kinematic model of the right leg.

The **pelvis module** includes a pelvis plate, centered at point P in Fig. S1.a, which is attached to the user's pelvis and can undergo both translational and rotational motion with respect to frame $\mathcal{F}$. This is achieved by a compliant spring-based connection between the fixed back of the pelvis module and the pelvis plate (see Wyss, 2019, for further details). The pelvis module functions as a series elastic actuator (SEA), with one actuated degree of freedom (P01-48x240 motor, NTI AG LinMot, Switzerland), enabling lateral movement relative to a fixed frame. To prevent hindering the natural lateral movement of the pelvis, the BWS is laterally actuated through a lead-screw mechanism parallel to the pelvis's actuated degree of freedom. Note that the other parts of the BWS remain unchanged with respect to their commercial version, i.e., allow for static and dynamic users' weight unloading.

Hip actuation is achieved through a closed-chain mechanism driven by two linear actuators per leg (P01-48x240 motors, LinMot, Switzerland). The two prismatic actuators per leg are located on a plane that can rotate around the x-axis of the global frame $\mathcal{O}$ (see Fig. S1.a). This configuration enables control of both hip flexion/extension and ab-/adduction, while maintaining a rigid joint rotation. This is achieved through a parallel closed-chain mechanism connected to the outputs of two joints (denoted by P_{int} and P_{ext} in Fig. S1.a) located at the distal end of the shafts of the hip prismatic actuators. On the other end, this closed-chain structure is attached to the thigh link of the orthosis via two perpendicular revolute joints (E and B in Fig. S1.a).

This mechanism converts the independent translational motions of the linear actuators into coordinated movements of the thigh link in the frontal and sagittal planes, as illustrated in Fig. S2.

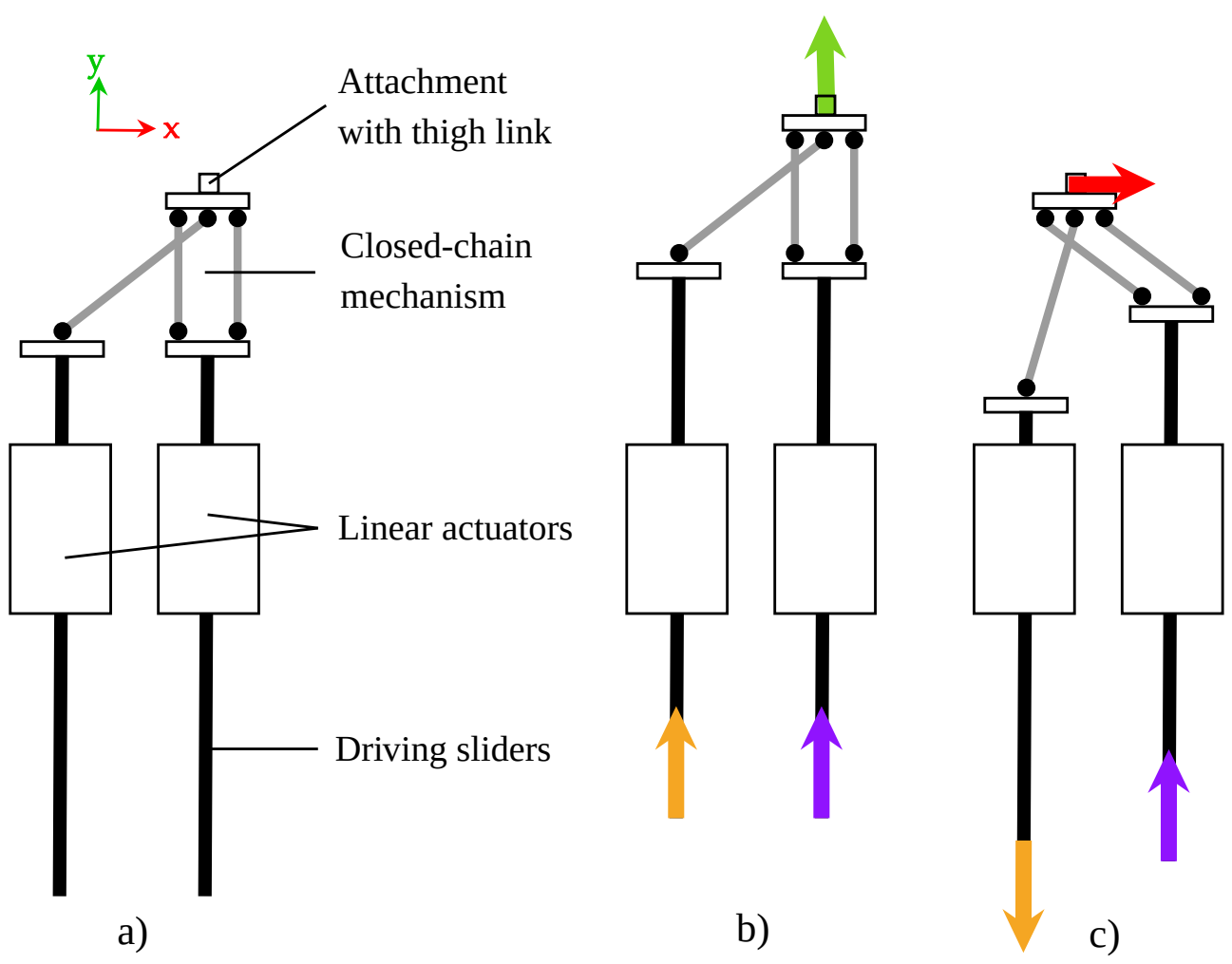

Figure S2: Top view of the right leg's closed-chain mechanism with the pelvis fixed in rotation, shown in different configurations: a) Neutral, b) Hip flexion/extension motion, and c) Hip ab-/adduction motion. Orange and purple arrows represent the movement of the linear actuators. Red and green arrows represent the resulting motion at the thigh.

In particular, this configuration enforces hip flexion/extension movements when the two linear actuators move in the same direction (Fig. S2.b), while ab-/adduction movements are produced by moving in opposite directions (Fig. S2.c). By allowing the linear actuators to rotate about the x-axis of the passive joint A, the system avoids overconstraining the leg.

In Fig. S1.a, the thigh rotational motion is represented by three rotational axes intersecting in H. Note that the hip internal/external rotation is mechanically constrained by the exoskeleton's structure and, aside from small contributions from pelvis rotation, remains approximately fixed. For completeness, Fig. S1.a also includes the **knee joint**, which is part of the original Lokomat® device. The knee joint is located at the distal end of the thigh link, connected to the closed-chain structure via a revolute joint (K) and provided with a built-in force sensor.

1.2 Exoskeleton kinematic model

We developed a closed-form analytical solution for the forward and inverse kinematics of the robot hip actuator module to be able to enforce personalized joint trajectories. Since the mechanism does not incorporate any sensors to measure the rotations of the hip joint at H, we determined the direct and inverse mapping between the actuators' joint coordinates (p_{int}, p_{ext}, Fig. S1.b) representing the shaft length of the two prismatic actuators of the hip module, and the space of hip joint coordinates (θ_{fl}, θ_{ab}, and θ_{ro}, Fig. S1.a), representing the hip flexion/extension, ab-/adduction, and internal/external rotation, respectively.

In the following paragraphs, we present the kinematic model and the process we followed for its definition, consisting of building the structure from both the perspective of the pelvis plate and the linear actuators to the spherical hip joint of the exoskeleton, and then closing the kinematic chain. Position and orientation of each joint will be expressed using homogeneous transformation matrices relative to the origin frame, $\mathcal{O}$. In the following equations, $\mathbf{T}_{\mathcal{X}}^{\mathcal{Y}}$ represents the translation vector from frame $\mathcal{Y}$ to frame $\mathcal{X}$, expressed in frame $\mathcal{Y}$ coordinates, and $\mathbf{R}_{\mathcal{X}}^{\mathcal{Y}}$ denotes the rotation matrix describing the orientation of frame $\mathcal{X}$ relative to frame $\mathcal{Y}$.

The hip module of the exoskeleton forms a closed kinematic chain that can be expressed by closing two branches of the loop, either at the hip joint H or at the upper hinge E of the parallel mechanism (see Fig. S1a). In the following, we derive the homogeneous positions of H and E from both closed-loop branches.

1.2.1 Top branch (O $\rightarrow \boldsymbol{H} \rightarrow \boldsymbol{M} \rightarrow \boldsymbol{E}$)

The pelvis plate pose is defined by its measured rotations α, β, γ, respect to fixed frame, around x,y and z axes respectively:

$$R_{\mathcal{P}}^{\mathcal{F}} = R_x(\alpha)R_y(\beta)R_z(\gamma), \qquad T_{\mathcal{P}}^{\mathcal{O}} = T_{\mathcal{F}}^{\mathcal{O}} + T_{\mathcal{P}}^{\mathcal{F}}. \tag{S1}$$

The hip joint H, rigidly mounted on the pelvis plate, is located at a calibrated offset $T_{\mathcal{H}}^{\mathcal{P}}$ to be adaptable to accommodate different user sizes:

$$T_{\mathcal{H}}^{\mathcal{O}}\big|_{\text{top}} = T_{\mathcal{P}}^{\mathcal{O}} + R_{\mathcal{P}}^{\mathcal{F}} T_{H}^{P}. \tag{S2}$$

where the index *top* indicates the propagation along the top branch. The hip orientation in O is given by

$$R_{\mathcal{H}}^{\mathcal{O}} = R_{\mathcal{P}}^{\mathcal{F}} R_{\mathcal{H}}^{\mathcal{P}}, \qquad R_{\mathcal{H}}^{\mathcal{P}} = R_x(\theta_{fl})R_y(\theta_{ab})R_z(\theta_{ro}). \tag{S3}$$

Propagating along the thigh yields

$$T^{\mathcal{O}}_{\mathcal{E}}\big|_{\text{top}} = T^{\mathcal{O}}_{\mathcal{H}}\big|_{\text{top}} + R^{\mathcal{O}}_{\mathcal{H}} \begin{bmatrix} 0 \\ -l_n \\ -l_m \end{bmatrix}. \tag{S4}$$

1.2.2 Bottom branch ($\boldsymbol{O} \rightarrow \boldsymbol{E} \rightarrow \boldsymbol{B} \rightarrow \boldsymbol{H}$)

The actuator plane A is rotated by θ_A about the x-axis:

$$R^{\mathcal{O}}_{\mathcal{A}} = R_x(\theta_A). \tag{S5}$$

The distal ends of the linear actuators are:

$$T^{\mathcal{A}}_{P_{\text{ext}}} = \begin{bmatrix} \frac{l_c}{2} \\ p_{\text{ext}} + d \\ 0 \end{bmatrix}, \quad T^{\mathcal{A}}_{P_{\text{int}}} = \begin{bmatrix} -\frac{l_c}{2} \\ p_{\text{int}} + d \\ 0 \end{bmatrix}. \tag{S6}$$

With link orientations $R^{\mathcal{A}}_{P_{\text{ext}}E} = R_z(\theta_1)$ and $R^{\mathcal{A}}_{P_{\text{int}}E} = R_z(-\theta_2)$, the hinge E satisfies:

$$T^{\mathcal{A}}_{\mathcal{E}} = T^{\mathcal{A}}_{P_{\text{ext}}} + R^{\mathcal{A}}_{P_{\text{ext}}E} \begin{bmatrix} 0 \\ l_1 \\ 0 \end{bmatrix} = T^{\mathcal{A}}_{P_{\text{int}}} + R^{\mathcal{A}}_{P_{\text{int}}E} \begin{bmatrix} 0 \\ l_2 \\ 0 \end{bmatrix}. \tag{S7}$$

Multiplying Eq. S7 by $\mathbf{R}^{O}_{A}$, the position of the revolute joint E can be obtained:

$$T^{\mathcal{O}}_{\mathcal{E}}\big|_{\text{bot}} = R^{\mathcal{O}}_{\mathcal{A}} T^{\mathcal{A}}_{\mathcal{E}} = \begin{bmatrix} \frac{l_c}{2} - l_1 \sin\theta_1 \\ \cos\theta_A(p_{\text{ext}} + d + l_1 \cos\theta_1) \\ \sin\theta_A(p_{\text{ext}} + d + l_1 \cos\theta_1) \end{bmatrix}. \tag{S8}$$

where the index *bot* indicates the propagation through the bottom branch. Propagating further along the bottom branch:

$$T^{\mathcal{O}}_{\mathcal{B}} = T^{\mathcal{O}}_{\mathcal{E}}\big|_{\text{bot}} + R^{\mathcal{O}}_{\mathcal{A}} R_x(\theta_E) \begin{bmatrix} 0 \\ l_n \\ 0 \end{bmatrix}, \tag{S9}$$

$$T^{\mathcal{O}}_{\mathcal{H}}\big|_{\text{bot}} = T^{\mathcal{O}}_{\mathcal{B}} + R^{\mathcal{O}}_{\mathcal{A}} R_x(\theta_E) R_y(\theta_B) \begin{bmatrix} 0 \\ 0 \\ l_m \end{bmatrix}. \tag{S10}$$

1.2.3 Loop-closure conditions

To solve for the unknown variables, the loop-closure equations are formulated by equating the two branches of the mechanism. The kinematic loop can be closed either at the hip joint H or at the upper

hinge E of the parallel mechanism:

$$\text{Closure at } H: \qquad T_{\mathcal{H}}^{\mathcal{O}}\big|_{\text{top}} = T_{\mathcal{H}}^{\mathcal{O}}\big|_{\text{bot}}, \tag{S11}$$

$$\text{Closure at } E: \qquad T_{\mathcal{E}}^{\mathcal{O}}\big|_{\text{top}} = T_{\mathcal{E}}^{\mathcal{O}}\big|_{\text{bot}}. \tag{S12}$$

Both systems of equations yield a valid solution to the closed-loop kinematics. The first formulation, closed at H, is particularly convenient for *forward kinematics* (joint $\rightarrow$ actuator space), whereas the second, closed at E, is more convenient for *inverse kinematics* (actuator $\rightarrow$ joint space).

The kinematic model of the mechanism was validated through an experiment performed by controlling the right leg of the exoskeleton in the execution of pre-recorded trajectories. Joint positions and angles obtained from the model were compared to those obtained from an OptiTrack™ motion capture system.

1.3 Extra Details of Kinematic Model Calculations

α, β, and γ denote the rotation angles of the pelvis frame $\mathcal{P}$ with respect to the fixed frame $\mathcal{F}$ about the x, y, and z axes of the fixed frame, respectively, corresponding to roll, pitch, and yaw in an extrinsic X–Y–Z rotation sequence. The corresponding rotation matrices are:

$$R_x(\alpha) = \begin{bmatrix} 1 & 0 & 0 \\ 0 & \cos\alpha & -\sin\alpha \\ 0 & \sin\alpha & \cos\alpha \end{bmatrix}, \quad R_y(\beta) = \begin{bmatrix} \cos\beta & 0 & \sin\beta \\ 0 & 1 & 0 \\ -\sin\beta & 0 & \cos\beta \end{bmatrix}, \quad R_z(\gamma) = \begin{bmatrix} \cos\gamma & -\sin\gamma & 0 \\ \sin\gamma & \cos\gamma & 0 \\ 0 & 0 & 1 \end{bmatrix}. \tag{S13}$$

The hip joint H is modeled as a 3-DoF rotational joint. The joint coordinates θ_{fl}, θ_{ab}, and θ_{ro} represent flexion/extension, abduction/adduction, and internal/external rotation, respectively, defined as extrinsic rotations about the x, y, and z axes of the pelvis frame $\mathcal{P}$. The corresponding rotation matrices are

$$R_x(\theta_{fl}) = \begin{bmatrix} 1 & 0 & 0 \\ 0 & \cos\theta_{fl} & -\sin\theta_{fl} \\ 0 & \sin\theta_{fl} & \cos\theta_{fl} \end{bmatrix}, \quad R_y(\theta_{ab}) = \begin{bmatrix} \cos\theta_{ab} & 0 & \sin\theta_{ab} \\ 0 & 1 & 0 \\ -\sin\theta_{ab} & 0 & \cos\theta_{ab} \end{bmatrix}, \quad R_z(\theta_{ro}) = \begin{bmatrix} \cos\theta_{ro} & -\sin\theta_{ro} & 0 \\ \sin\theta_{ro} & \cos\theta_{ro} & 0 \\ 0 & 0 & 1 \end{bmatrix}. \tag{S14}$$

1.4 Validation of the kinematic model

To validate the kinematic model of the mechanism, we performed an experiment with the right leg of the exoskeleton. After recording a healthy subject walking in the exoskeleton, while set in transparent mode, the obtained gait pattern was commanded to the exoskeleton (without, this time, any participant wearing it) in rigid position control mode. The computed kinematics were compared against a ground-truth motion capture system.

During the execution of the pre-recorded trajectory, we recorded data from the device motor encoders and optical sensors, as well as from an OptiTrack^{TM} motion capture system. The motion capture system consisted of four Prime^{x} 13W cameras, which measured the position and orientation of the key components: hip linear actuators, pelvis fixed frame, pelvis back plate, and thigh link.

To evaluate the **forward kinematics model**, we used the robot sensor data as inputs, including pelvis pose and prismatic joint positions recorded during the experiment. These inputs were fed into a Simulink/SimMechanics model of the system using MATLAB® Simscape Multibody (R2021b, MathWorks, USA) to compute the corresponding hip joint angles, namely the flexion/extension, abduction/adduction, and internal/external rotation (see Fig. S3 for a screenshot).

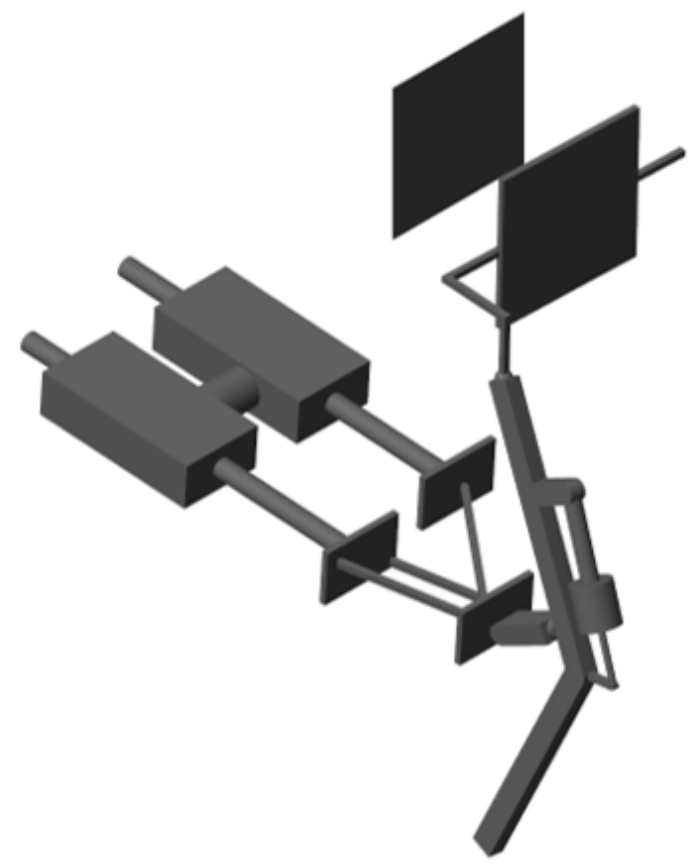

Figure S3: Screenshot of the mechanism Simscape model.

The simulated joint angles were then compared against those measured directly via the motion capture system to assess model accuracy (see Fig.S4). Accuracy was assessed by computing maximum and root-mean-square errors (RMSE) between the recorded and simulation data. The maximum error between the motion capture values and those calculated from the forward kinematics was $2.97°$ in hip flexion/extension, $0.76°$ in hip abduction/adduction and $0.41°$ in hip rotation. The RMSE was $2.2°$ in hip flexion/extension, $0.32°$ in hip abduction/adduction, and $0.78°$ in hip rotation.

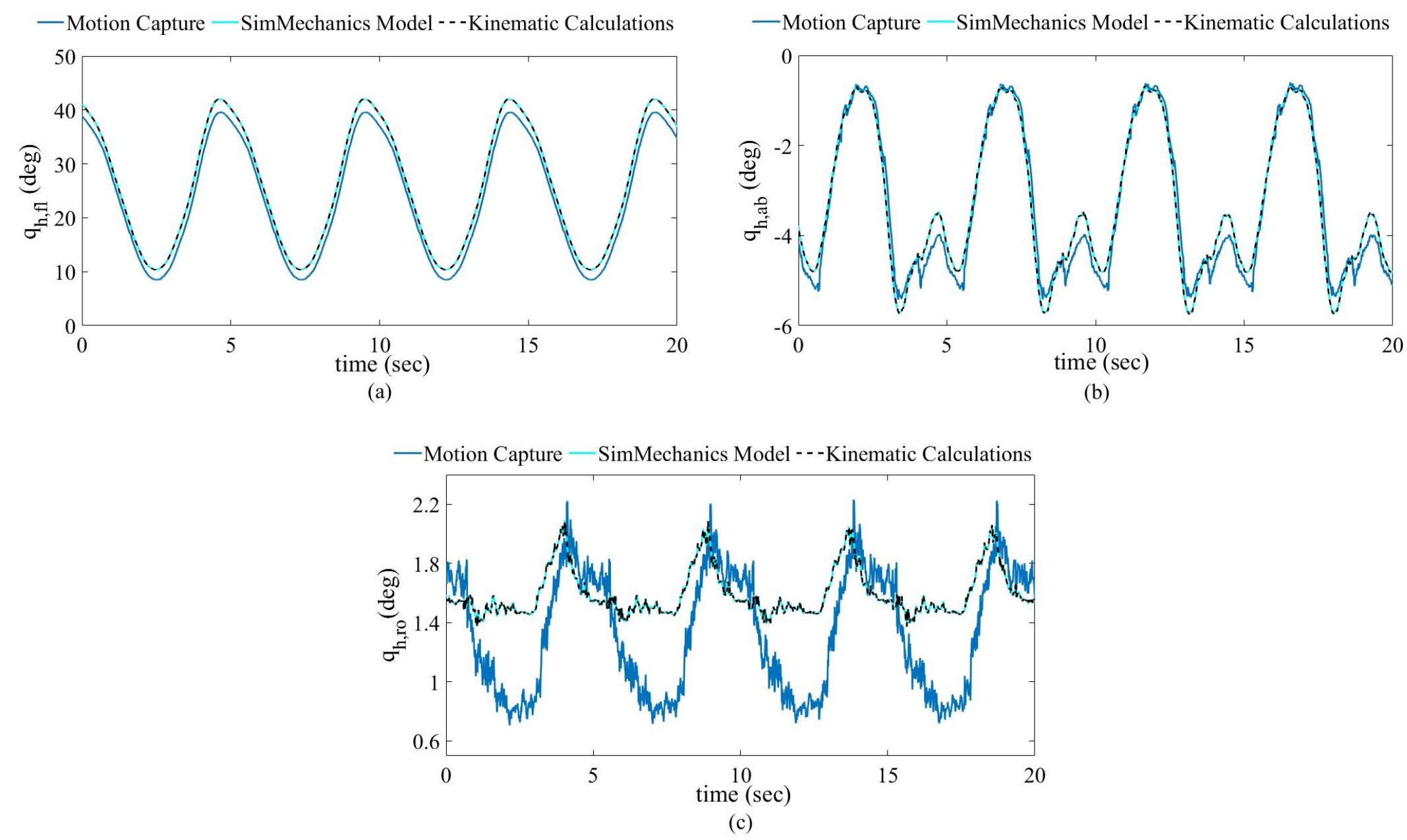

Figure S4: Results from the forward kinematics validation. (a) Hip flexion/extension. (b) Hip abduction/adduction. (c) Hip rotation.

To validate the **inverse kinematics** mapping, we used hip joint angles measured via motion capture as inputs and computed the corresponding prismatic joint positions. These model outputs were then compared to the encoder readings of the linear actuators (see Fig.S5). The maximum error for both internal

and external actuators was below one centimeter, with root mean square errors (RMSE) of $0.78\,\mathrm{cm}$ and $0.77\,\mathrm{cm}$, respectively.

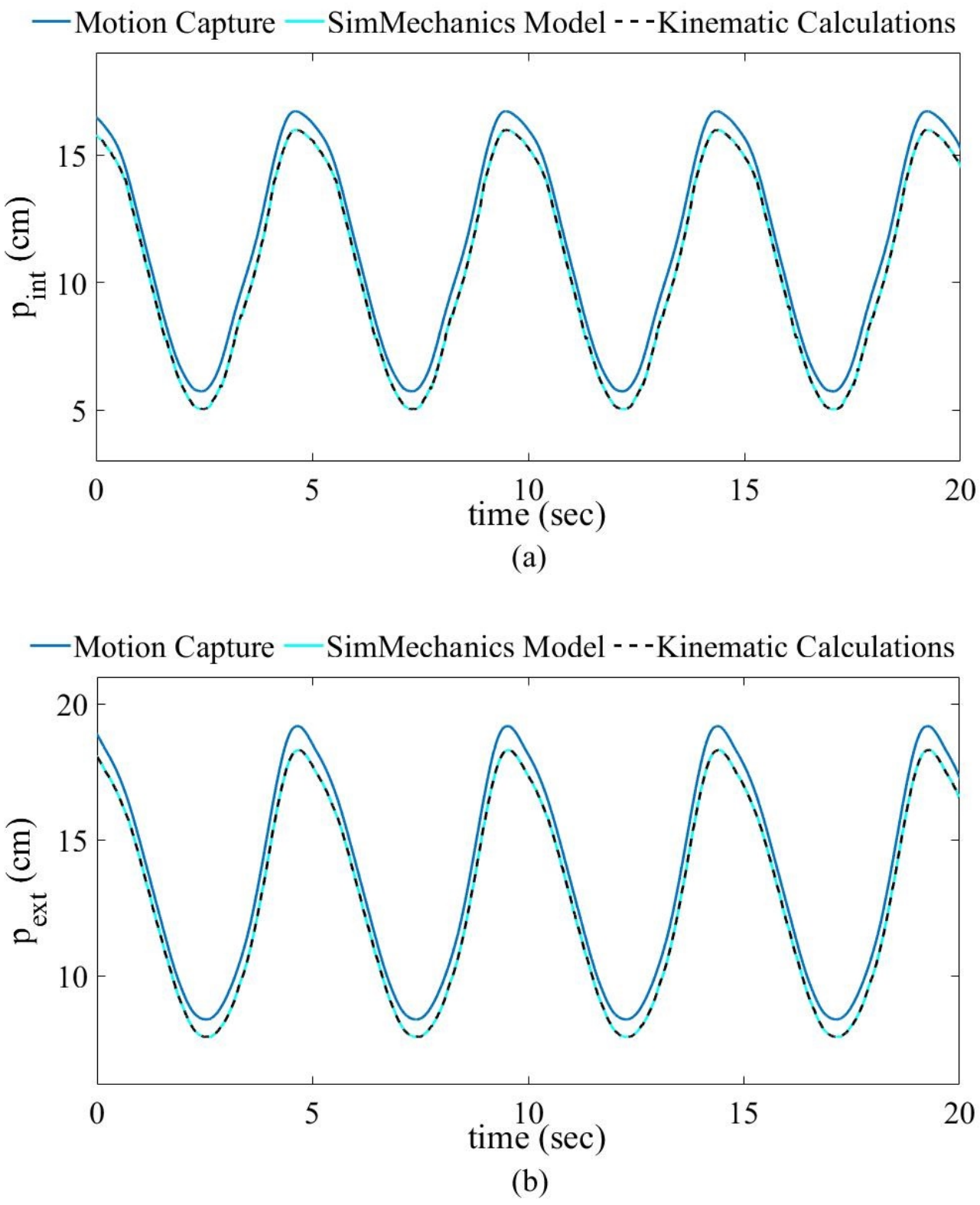

Figure S5: Results from the inverse kinematics. (a) Position of internal prismatic actuator. (b) Position of external prismatic actuator

2 REGRESSION EQUATIONS OF JOINT TRAJECTORIES

The regression coefficients derived for the different joint trajectories and their corresponding key events are listed as follows: hip abduction/adduction in Table S1, hip flexion/extension in Table S2, knee flexion/extension in Table S3, and lateral pelvis movement in Table S4.

Table S1. Regression equations for the parameter values of the key events of hip abduction/adduction.

**Hip abduction/adduction**								
Key event	Parameter	β_c (Intercept)	β_v (Speed)	β_{v^2} (Speed^2)	β_h (Height)	β_w (Weight)	β_a (Age)	β_s (Gender)
Heel contact	Timing (t)	0	-	-	-	-	-	-
	Angle (θ)	0.0400	-	-	-	-	-	-
	Vel. ($\dot{\theta}$)	0.0222	-	0.0075	-	-	-	-0.0343
	Acc. ($\ddot{\theta}$)	0.0390	-	0.0042	-	-	-	-0.0182
Max. stance	Timing (t)	35.8245	-	-	-10.0179	-	-	0.7653
	Angle (θ)	15.0676	-	-	-8.2105	0.0568	-	-1.3062
	Vel. ($\dot{\theta}$)	0	-	-	-	-	-	-
	Acc. ($\ddot{\theta}$)	-0.0914	-	-	-	-	0.0006	0.0108
Max. $\dot{y}$ stance	Timing (t)	41.0000	-	-	-	-	-	-
	Angle (θ)	17.5024	-	-	-12.0096	0.0790	-	-0.5726
	Vel. ($\dot{\theta}$)	-0.0130	-	-	-	-	-	-
	Acc. ($\ddot{\theta}$)	0	-	-	-	-	-	-
Min. $\dot{y}$ swing	Timing (t)	58.3550	-	-	-	-	-	-
	Angle (θ)	-4.0730	-	-	-	-	0.0479	-
	Vel. ($\dot{\theta}$)	-2.3595	-	-0.0138	1.4393	-0.0105	0.0034	-
	Acc. ($\ddot{\theta}$)	0	-	-	-	-	-	-
Min. swing	Timing (t)	74.1734	-1.7955	-	-	-	-	-
	Angle (θ)	-19.8236	-	-	8.9280	-0.0598	0.0725	-
	Vel. ($\dot{\theta}$)	0	-	-	-	-	-	-
	Acc. ($\ddot{\theta}$)	0.2778	-	-	-0.1289	0.0013	-0.0008	-0.0190
Max. $\dot{y}$ swing	Timing (t)	69.8878	-	-	-	0.0891	0.0331	-
	Angle (θ)	-5.2261	-	-	-	-	0.0444	-
	Vel. ($\dot{\theta}$)	0.4375	-0.0366	-	-	0.0037	-0.0030	-0.0956
	Acc. ($\ddot{\theta}$)	0	-	-	-	-	-	-

Note: “-” indicates that a parameter has no significant impact on the regression model. The regression equations were generated using data from all 42 subjects. By default, heel strike timing is set to zero (% of the gait cycle), as are the minimum and maximum values for joint angle and angular velocity.

Table S2. Regression equations for the parameter values of the key events of hip flexion/extension.

**Hip flexion/extension**								
Key Event	Parameter	β_c (Intercept)	β_v (Speed)	β_{v^2} (Speed^2)	β_h (Height)	β_w (Weight)	β_a (Age)	β_s (Gender)
Heel contact	Timing (t)	0	-	-	-	-	-	-
	Angle (θ)	24.4160	-	-	-	-	0.0830	-
	Vel. ($\dot{\theta}$)	-0.8169	0.1166	-	0.1915	-	-	-
	Acc. ($\ddot{\theta}$)	-0.3435	0.0775	-	-	-	-	0.0147
Max. stance[a]	Timing (t)	15.6875	-	-	-5.6864	-	-	-
	Angle (θ)	30.1610	-	-	-	-	-	-
	Vel. ($\dot{\theta}$)	0	-	-	-	-	-	-
	Acc. ($\ddot{\theta}$)	-1.0905	-	-	0.7260	-0.0043	-	-
t=50% Stance	Timing (t)	31.4315	-2.3754	0.3413	-	-	-	-
	Angle (θ)	5.4895	-	-	-	-	0.0868	-
	Vel. ($\dot{\theta}$)	-1.2312	-0.1953	-	0.6511	-	-	-
	Acc. ($\ddot{\theta}$)	0.0601	-	-0.0017	-	-	-0.0005	-
Min.	Timing (t)	63.4402	-4.7863	0.6803	-	-	-	-
	Angle (θ)	-4.5359	-	-	-	-	-	1.9210
	Vel. ($\dot{\theta}$)	0	-	-	-	-	-	-
	Acc. ($\ddot{\theta}$)	0.1171	0.0117	-	-	-	-	-
Max. $\dot{y}$ swing	Timing (t)	75.3750	-9.3496	1.6596	-	0.0719	-	-1.2761
	Angle (θ)	8.4660	-	-	-	-	-	-
	Vel. ($\dot{\theta}$)	3.2209	0.0980	-	-1.2215	0.0081	-0.0033	-0.0829
	Acc. ($\ddot{\theta}$)	0	-	-	-	-	-	-
Max. swing	Timing (t)	90.6226	-1.0169	-	-	-	0.0474	-
	Angle (θ)	61.8406	2.0764	-	-21.7837	-	-	1.4937
	Vel. ($\dot{\theta}$)	0	-	-	-	-	-	-
	Acc. ($\ddot{\theta}$)	-0.1080	-	-	-	-	-	-

[a] This key event is excluded from the spline fitting procedure due to its relevance only for walking speeds exceeding 3.5 kph, whereas the developed regression model focuses on speeds up to 3.2 kph.

Table S3. Regression equations for the parameter values of the key events of knee flexion/extension.

**Knee flexion/extension**								
Key Event	Parameter	β_c (Intercept)	β_v (Speed)	β_{v^2} (Speed^2)	β_h (Height)	β_w (Weight)	β_a (Age)	β_s (Gender)
Heel contact	Timing (t)	0	-	-	-	-	-	-
	Angle (y)	-18.4403	-	-0.3887	10.1799	-	0.1908	-
	Vel. ($\dot{y}$)	-0.8710	0.7016	-0.0875	-	-	-	0.0664
	Acc. ($\ddot{y}$)	-0.5245	0.2494	-	-	-	-	0.0490
Max. stance	Timing (t)	11.643	-	-	-	-	-	-
	Angle (y)	-2.2525	-	0.4534	-	-	0.2254	2.1273
	Vel. ($\dot{y}$)	0	-	-	-	-	-	-
	Acc. ($\ddot{y}$)	-0.3389	-	-	0.3752	-0.0074	-0.0017	0.0743
Min. stance	Timing (t)	32.4633	-	-	-	-	0.1521	1.9152
	Angle (y)	16.7077	-	-	-	-0.2192	-	3.2653
	Vel. ($\dot{y}$)	0	-	-	-	-	-	-
	Acc. ($\ddot{y}$)	0.1067	0.0202	-	-0.0980	0.0012	-	-
Max. $\dot{y}$ swing	Timing (t)	77.1875	-7.9539	1.1427	-	-	-	-0.4295
	Angle (y)	37.1826	1.4471	-	-	-0.0980	-	-
	Vel. ($\dot{y}$)	3.2140	-	-	-	0.0061	-	-0.1909
	Acc. ($\ddot{y}$)	0	-	-	-	-	-	-
Max. swing	Timing (t)	81.719	-5.0453	0.8888	-	-	-	-
	Angle (y)	52.1027	4.7823	-	-	-0.1198	-	-
	Vel. ($\dot{y}$)	0	-	-	-	-	-	-
	Acc. ($\ddot{y}$)	-0.9725	0.0768	-	0.1608	-	0.0014	0.0297
Min. $\dot{y}$ swing	Timing (t)	88.3116	-	0.1551	-	-	-	-
	Angle (y)	17.7490	-	-	-	-	0.1429	-
	Vel. ($\dot{y}$)	-3.9013	-0.4602	-	-	0.0143	0.0098	-
	Acc. ($\ddot{y}$)	0	-	-	-	-	-	-

Table S4. Regression equations for the parameter values of the key events of lateral pelvis movement.

**Lateral pelvis movement**								
Key Event	Parameter	β_c (Intercept)	β_v (Speed)	β_{v^2} (Speed^2)	β_h (Height)	β_w (Weight)	β_a (Age)	β_s (Gender)
Max. $\dot{y}$	Timing (t)	12.7882	-	-	-	-	-0.0266	-
	Angle (y)	6.9961	-	-	-	-	-0.0735	-1.7287
	Vel. ($\dot{y}$)	7.1648	-0.7182	-	-2.0033	0.0210	-0.0142	-
	Acc. ($\ddot{y}$)	0	-	-	-	-	-	-
Max.	Timing (t)	26.1353	-	-	5.6096	-	-	-
	Angle (y)	60.1065	-9.2432	-	-	-	-	-
	Vel. ($\dot{y}$)	0	-	-	-	-	-	-
	Acc. ($\ddot{y}$)	-0.1353	-	0.0031	-	-	-	-
Min. $\dot{y}$	Timing (t)	62.9191	-	-	-	-	-0.0243	-
	Angle (y)	4.2084	-	-	-	-0.1631	0.0582	3.1179
	Vel. ($\dot{y}$)	-4.7921	0.7730	-	-	-	-	-
	Acc. ($\ddot{y}$)	0	-	-	-	-	-	-
Min.	Timing (t)	75.8100	-	-	5.6217	-	-	-
	Angle (y)	-60.1309	9.2839	-	-	-	-	-
	Vel. ($\dot{y}$)	0	-	-	-	-	-	-
	Acc. ($\ddot{y}$)	0.0387	-	-0.0018	-	0.0014	-	-0.0212

3 QUANTITATIVE DATA RESULTS

The analysis of the exoskeleton's data recordings in terms of Mean Absolute Error (MAE) of actuator positions is reported in Table S5.

Table S5. Linear mixed model results for position error: *Mean Absolute Error (MAE)* of the different actuators. BWS: Body Weight Support actuator.

Variable	Pelvis Prismatic Actuator: MAE - Position (mm)				BWS Prismatic Actuator: MAE - Position (mm)			
	**Estimate (β)**	**Std. Error**	**t value**	**p-value**	**Estimate (β)**	**Std. Error**	**t value**	**p-value**
(Intercept)	4.05	0.36	11.29	**2.61E-11*****	2.54	0.21	11.96	**1.48E-11*****
Predicted pattern	1.46	0.39	3.78	**8.67E-04****	0.44	0.22	2.02	0.060
Random pattern	0.58	0.39	1.50	0.147	0.71	0.22	3.24	**0.005****
2nd Trial	0.53	0.39	1.37	0.181	0.23	0.22	1.03	0.317
3rd Trial	0.14	0.39	0.37	0.713	0.05	0.22	0.22	0.827
**Variable**	Right Outer Prismatic Actuator: MAE - Position (mm)				Right Inner Prismatic Actuator: MAE - Position (mm)			
	**Estimate (β)**	**Std. Error**	**t value**	**p-value**	**Estimate (β)**	**Std. Error**	**t value**	**p-value**
(Intercept)	8.35	0.15	56.41	**8.77E-28*****	5.96	0.37	15.99	**1.23E-14*****
Predicted pattern	-0.53	0.14	-3.85	**0.001****	-0.98	0.40	-2.45	**0.022***
Random pattern	-1.13	0.14	-8.17	**4.20E-07*****	-0.12	0.40	-0.30	0.770
2nd Trial	-0.20	0.14	-1.48	0.157	-0.92	0.40	-2.30	**0.030***
3rd Trial	-0.18	0.14	-1.32	0.205	-0.32	0.40	-0.81	0.425
**Variable**	Left Outer Prismatic Actuator: MAE - Position (mm)				Left Inner Prismatic Actuator: MAE - Position (mm)			
	**Estimate (β)**	**Std. Error**	**t value**	**p-value**	**Estimate (β)**	**Std. Error**	**t value**	**p-value**
(Intercept)	8.48	0.15	58.43	**3.26E-28*****	5.95	0.38	15.83	**1.53E-14*****
Predicted pattern	-0.52	0.14	-3.84	**0.001****	-0.82	0.40	-2.04	0.053
Random pattern	-1.08	0.14	-7.95	**6.04E-07*****	-0.13	0.40	-0.32	0.753
2nd Trial	-0.19	0.14	-1.36	0.193	-0.94	0.40	-2.33	**0.028***
3rd Trial	-0.19	0.14	-1.38	0.186	-0.35	0.40	-0.87	0.394
**Variable**	Right Knee Revolute Actuator: MAE - Position (deg)				Left Knee Revolute Actuator: MAE - Position (deg)			
	**Estimate (β)**	**Std. Error**	**t value**	**p-value**	**Estimate (β)**	**Std. Error**	**t value**	**p-value**
(Intercept)	2.87	0.20	14.15	**2.95E-12*****	3.06	0.27	11.46	**4.91E-09*****
Predicted pattern	-0.14	0.16	-0.84	0.411	-0.02	0.17	-0.14	0.888
Random pattern	0.26	0.16	1.65	0.118	0.27	0.17	1.60	0.128
2nd Trial	-0.47	0.16	-2.96	**0.009****	-0.49	0.17	-2.94	**0.010***
3rd Trial	-0.48	0.16	-2.99	**0.009****	-0.25	0.17	-1.48	0.157

*($p < 0.05$), **($p < 0.01$), ***($p < 0.001$)

The comparison between patterns (Predicted, Random, and Personalized), averaged over participants, is reported in Tab.S6

Table S6. RMSE between gait pattern pairs. Values are reported as mean $\pm$ SD across participants. Pelvis, BWS, and hip actuator trajectories are reported in meters, while knee trajectories are reported in degrees.

Actuators	Predicted vs Standard	Predicted vs Random	Standard vs Random
Pelvis Prismatic [mm]	0.0438 ± 0.0030	0.0442 ± 0.0044	0.0440 ± 0.0143
BWS Prismatic [mm]	0.0438 ± 0.0030	0.0442 ± 0.0044	0.0440 ± 0.0143
Right Inner Prismatic [mm]	0.0838 ± 0.0049	0.0899 ± 0.0184	0.1059 ± 0.0281
Right Outer Prismatic [mm]	0.1151 ± 0.0066	0.1163 ± 0.0119	0.1296 ± 0.0291
Left Inner Prismatic [mm]	0.0843 ± 0.0049	0.0906 ± 0.0184	0.1058 ± 0.0281
Left Outer Prismatic [mm]	0.1152 ± 0.0067	0.1161 ± 0.0117	0.1294 ± 0.0291
Right Knee Revolute [deg]	25.22 ± 1.28	24.35 ± 2.20	26.11 ± 6.36
Left Knee Revolute [deg]	25.22 ± 1.27	24.36 ± 2.19	26.10 ± 6.36

4 QUESTIONNAIRES AND RESPONSES TO OPEN-ENDED QUESTIONS

4.1 User experience questionnaires

4.1.1 Questions

Tab.S7 reports a summary of the questions asked per category assessing the user experience.

Table S7. Perception questionnaire items.

Category	Questions
Interest/ Enjoyment (IMI)	I enjoyed doing this activity very much<br>I thought this was a boring activity<br>I thought the task was very interesting<br>The activity was fun to do
Passiveness	I remained passive during the activity<br>I resisted the movements of the exoskeleton<br>I allowed the exoskeleton to guide my lower limbs<br>I actively tried to control or influence the movements of the exoskeleton
Comfort	How comfortable were the movements of the exoskeleton?<br>How would you rate the comfort at the cuffs during movement?<br>How comfortable were the movements in the hips?<br>How comfortable were the movements in the knees?<br>How comfortable were the movements in the ankles/feet?<br>I felt physical strain during the activity<br>I felt like I was secure in the device
Naturalness	How natural did the movements of the exoskeleton feel?<br>How similar was the applied gait pattern compared to your own walking?<br>How smooth did the movements of the exoskeleton feel?<br>I felt the exoskeleton was pushing my limbs beyond my natural range.

4.1.2 Responses summary

Figure S6 presents the statistics of scores assigned to the various categories of the questionnaire.

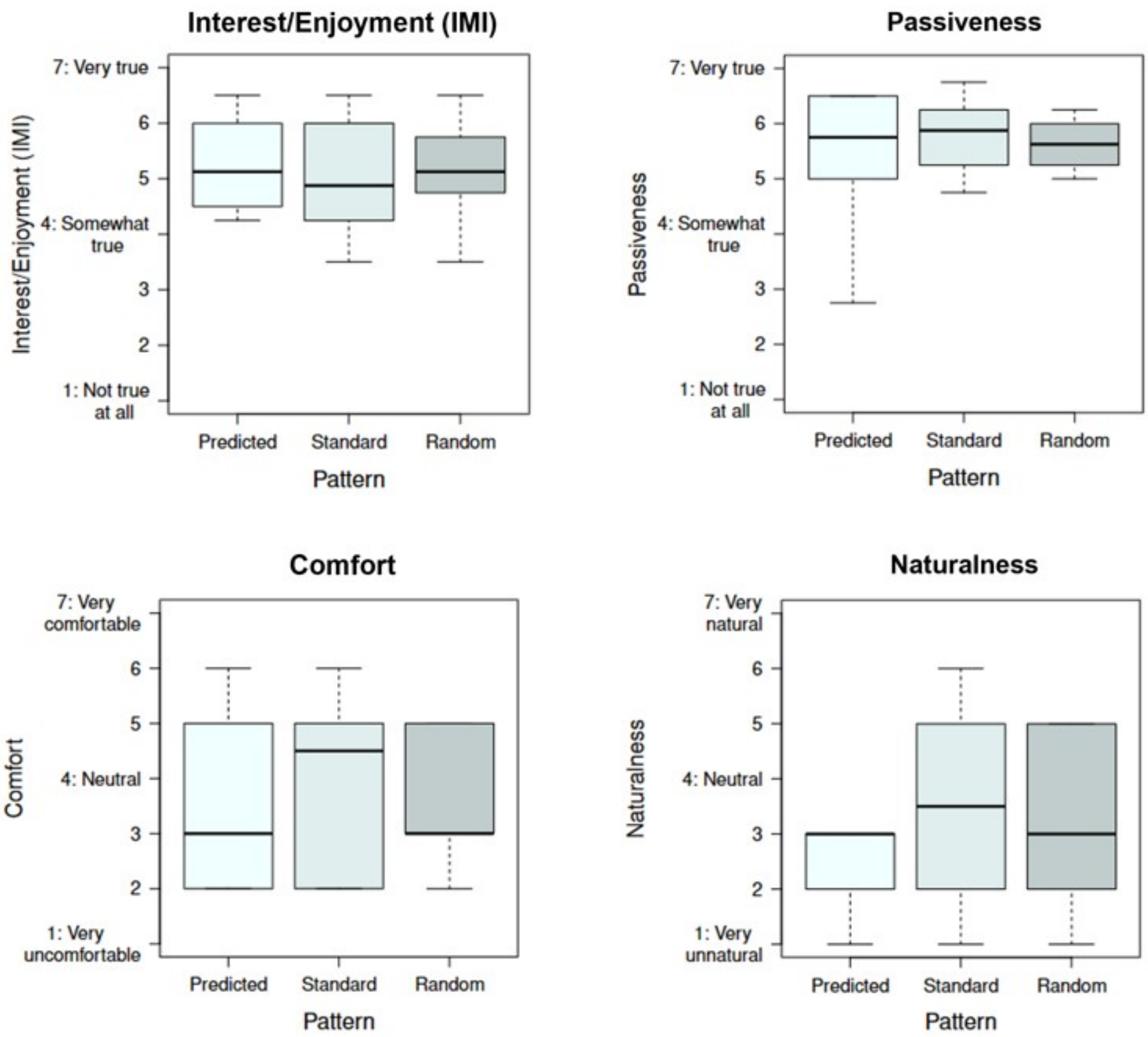

Figure S6: Scores attributed to the different categories of the questionnaire

4.1.3 Linear Mixed Models

Table S8. Linear mixed model results for user experience metrics.

**Enjoyment and Passiveness**								
	Enjoyment/Interest (IMI)[2]				Passiveness[2]			
	Estimate (β)	Std. Error	t value	p-value	Estimate (β)	Std. Error	t value	p-value
(Intercept)	5.01	0.30	16.58	**5.28E-10** ***	5.68	0.36	15.60	**2.37E-14** ***
Personalized	0.20	0.15	1.34	0.199	-0.30	0.36	-0.84	0.413
Random	0.13	0.15	0.85	0.409	-0.29	0.36	-0.81	0.429
2nd Trial	0.01	0.15	0.08	0.933	0.10	0.36	0.27	0.792
3rd Trial	0.02	0.15	0.12	0.907	0.10	0.36	0.28	0.781
**Comfort**								
	Overall Comfort[1]				Comfort at Cuffs[1]			
(Intercept)	3.29	0.52	6.31	**1.35E-06** ***	3.81	0.49	7.82	**2.88E-07** ***
Personalized	-0.75	0.49	-1.53	0.145	0.40	0.35	1.17	0.260
Random	-0.40	0.49	-0.83	0.419	0.41	0.35	1.20	0.249
2nd Trial	0.96	0.49	1.97	0.067	0.14	0.35	0.41	0.688
3rd Trial	1.43	0.49	2.94	**0.01** **	0.10	0.35	0.29	0.774
	Comfort at Hips[1]				Comfort at Knees[1]			
(Intercept)	4.39	0.63	7.02	**2.42E-07** ***	5.16	0.57	9.00	**2.72E-09** ***
Personalized	-0.26	0.58	-0.45	0.655	-0.15	0.53	-0.29	0.779
Random	-0.04	0.58	-0.07	0.945	-0.55	0.53	-1.03	0.320
2nd Trial	0.60	0.58	1.03	0.317	-0.45	0.53	-0.86	0.405
3rd Trial	0.22	0.58	0.38	0.705	0.06	0.53	0.11	0.911
	Comfort at Ankles/Feet[1]				Physical Strain[2]			
(Intercept)	4.05	0.53	7.64	**6.37E-08** ***	2.10	0.43	4.86	**6.14E-05** ***
Personalized	-0.44	0.47	-0.94	0.362	1.07	0.37	2.86	**0.011** *
Random	-0.28	0.47	-0.60	0.559	0.44	0.37	1.19	0.252
2nd Trial	0.17	0.47	0.36	0.722	0.44	0.37	1.19	0.252
3rd Trial	0.62	0.47	1.30	0.212	-0.26	0.37	-0.70	0.492
	Sense of Security in Device[2]							
(Intercept)	5.15	0.45	11.44	**8.22E-10** ***				
Personalized	-0.13	0.32	-0.41	0.687				
Random	0.22	0.32	0.69	0.497				
2nd Trial	0.22	0.32	0.69	0.497				
3rd Trial	0.54	0.32	1.67	0.114				
**Naturalness**								
	Naturalness of Movements[1]				Similarity to Own Way of Walking[1]			
(Intercept)	2.68	0.52	5.20	**2.25E-05** ***	2.56	0.51	4.98	**4.01E-05** ***
Personalized	-0.71	0.49	-1.45	0.165	-0.76	0.51	-1.50	0.154
Random	-0.17	0.49	-0.35	0.728	-0.58	0.51	-1.14	0.272
2nd Trial	1.28	0.49	2.64	0.018	1.24	0.51	2.45	0.026
3rd Trial	1.35	0.49	2.79	**0.013** **	1.82	0.51	3.59	**0.002** **
	Smoothness of Movements[1]				Limbs Pushed Beyond Natural Range[2]			
(Intercept)	4.61	0.40	11.42	**2.09E-10** ***	2.00	0.40	5.04	**5.21E-05** ***
Personalized	0.16	0.31	0.52	0.614	-0.09	0.32	-0.29	0.777
Random	0.11	0.31	0.35	0.728	-0.42	0.32	-1.35	0.197
2nd Trial	0.11	0.31	0.35	0.728	-0.24	0.32	-0.77	0.453
3rd Trial	0.49	0.31	1.58	0.134	-0.33	0.32	-1.06	0.306

*($p < 0.05$), **($p < 0.01$), ***($p < 0.001$)

[1] 7-point Likert Scale: (1) Very unnatural/uncomfortable/dissimilar - (4) Neutral - (7) Very natural/comfortable/similar. [2] 7-point Likert Scale: (1) Not true at all - (4) Somewhat true - (7) Very true

4.2 Open ended questions

The answers of the open-ended questions are organized into the tables below: Table S9 contains responses related to discomfort and unusual sensations; Table S10 contains responses related to aspects influencing comfort or discomfort; and Table S11 includes additional thoughts or comments provided by participants.

Table S9. Participant responses regarding discomfort, physical strain, and unusual sensations experienced during walking with the robot, grouped by gait pattern.

n.	Standard	Personalized	Random
1	"I felt like there was some weight on my feet which made it difficult to remain passive. Discomfort level: 6/10."	"I felt discomfort with how much weight was applied on my feet. Because of this it was hard to remain passive. Discomfort level: 7/10."	"I felt some discomfort at the hips (6/10)."
2	"Cuffs at the ankles still uncomfortable as during the random pattern. Much less strain to keep foot position."	"Very similar to the standard pattern."	"Yes, the machine was too low while walking and my feet did not have room to go back. If the machine was higher, the ankle cuffs pressed too much against my calf. Keeping my feet horizontal was tiring."
3	"I still felt strain around the ankle cuffs seemingly pushing my calf muscles up, although less than in the previous trial. I also felt some chafing around the hip bone."	"Ankle cuffs keep pushing the muscles of my calves up. This creates a cramped feeling. It also felt as if the space between my thighs was larger than normal."	"This pattern resulted in the least chafing. However, excessive hip swaying began to hurt at the hip bone and the ankle cuffs started to chafe again."
4	–	–	"Yes, a slight discomfort in the knees."
5	"Yes, particularly uncomfortable on the hips."	"Slight discomfort at ankles (especially left)."	"Light discomfort on calf cuff. Medium discomfort at ankle and knee."
6	–	"My muscles got a bit sore since you relax most muscles except for the calves due to the ankle movements."	"No, this was much better compared to the first two gait patterns, although I may also have become accustomed to it."
7	–	–	–
8	"Ankle cuffs a bit uncomfortable."	"Ankle cuffs uncomfortable."	"Right ankle cuff was sometimes a bit uncomfortable."
9	"I felt slight discomfort in my ankles when walking with the exoskeleton. The joints were moved into an unnatural position."	"I experienced discomfort around the cuffs because my legs were pushed beyond the natural range of motion."	"No physical strain."
10	–	"Mild discomfort on the outer part of my feet when landing after every step."	–

Table S10. Participant responses regarding aspects of the gait pattern that contributed to comfort or discomfort, grouped by gait pattern.

n.	Standard	Personalized	Random
1	"My feet were moving too close to each other, which was unnatural for me."	"Again I felt like my feet were too close to each other during the trial."	"I felt like my feet were a bit too close to each other when walking, which was unnatural for me."
2	"Acceleration still a bit abrupt." (referring to the random pattern)	"I felt the accelerations were less abrupt compared to the other two conditions."	"I felt the forward acceleration phase was unnaturally fast."
3	"My hips were still swaying quite far sideways. This time it was less problematic because the movement was smaller and my body partially countered the weight distribution. This made it feel less like my body was being thrown left to right by the hips."	"I felt my hips were swaying outside the range of my legs (or where my feet were positioned on the ground), creating a sensation as if I would fall if I had to walk like this outside the device."	"There was once again excessive hip swaying, the worst of the three conditions."
4	–	–	"Not sure about the source, but my knees felt rather stiff."
5	"The trajectory was moving my waist left and right, making the hip movement less pleasant and natural."	"My left leg was pushed a bit towards the right, making the left ankle act somewhat shaky at the end of the motion."	"My legs were positioned more forward than their natural position. This implied better movement for the hip but worse for the knee. It was difficult to raise the tip of the foot enough."
6	"The gait pattern was somewhat counterintuitive since it felt like I was not walking in a straight line."	"The lower-limb movement was okay, but my feet were too close to the centre. Also, the hips were moved too much towards the outside, which was uncomfortable."	"Now I know why it was uncomfortable: my feet were too far apart, so I had to compensate with my hips, which was uncomfortable."
7	"This time my legs seemed to move from the outside to the inside when extending the leg forward. This felt more similar to my usual gait."	"I felt like the legs were sometimes moving from the inside to the outside during forward leg extension. Depending on whether I was leaning to the right or left, it felt asymmetrical."	"The hips were moving less from side to side, which made it more comfortable. However, the legs moved more to the outside during extension, which felt somewhat unnatural."
8	"Also a lot of lateral hip movement."	"Also lateral hip movement [uncomfortable]."	"Too much lateral movement of my hips felt funny."
9	"The gait pattern moved my feet in an unnatural way during heel strike."	"My discomfort was high because there was an unnatural motion for my legs to walk with."	"Comfort in my ankles was higher due to smaller movements and slower jerking motions compared to the first trial (standard pattern)."
10	"I felt that the steps were wider and that the trajectory followed by the foot from start to end of the step was straighter, making it more similar to my gait."	"The trajectory of my knees and ankles felt unnatural because it moved in and out before foot contact."	"The ankles were pushed inward, which felt unnatural. The knee motion also showed some overshoot before foot contact."

Table S11. Participant responses regarding additional thoughts or comments, grouped by gait pattern.

n.	Standard	Personalized	Random
1	–	–	–
2	–	–	–
3	–	–	"1) Despite the points above, the leg movements and timing felt the most natural during this trial. The legs felt too spread apart and the hip swaying made it feel as if I would fall, but the overall leg movement felt surprisingly natural. 2) Sometimes the machine movements felt a little odd during this trial, as if it would briefly speed up. This happened only once or twice, so it may have been nothing, but I noticed it."
4	"The hip movement felt a bit unusual, perhaps slightly exaggerated."	"It was not discomfort per se, but the hip movement felt over-exaggerated."	–
5	–	"I felt that my weight was placed more on the right leg, like a 60–40 distribution."	–
6	–	"Because of the unnatural movements, I really had to focus on what I was doing instead of letting go and walking naturally."	–
7	–	–	–
8	–	–	–
9	"The rest of the gait pattern felt natural."	"I think the unnatural movements were contributing to the strain/discomfort around the cuffs, but I am not sure."	–
10	–	–	–
